# Bacteriophages, gut bacteria, and microbial pathways interplay in cardiometabolic health

**DOI:** 10.1016/j.celrep.2024.113728

**Published:** 2024-01-31

**Authors:** Daniel Kirk, Ricardo Costeira, Alessia Visconti, Mohammadali Khan Mirzaei, Li Deng, Ana M. Valdes, Cristina Menni

**Affiliations:** 1 Department of Twin Research & Genetic Epidemiology, King’s College London, St Thomas Hospital, Westminster Bridge Road, London SE1 7EH, UK; 2 Center for Biostatistics, Epidemiology, and Public Health, Department of Clinical and Biological Sciences, University of Turin, Turin, Italy; 3 Institute of Virology, Helmholtz Centre Munich - German Research Centre for Environmental Health, 85764 Neuherberg, Germany; 4 School of Life Sciences, Technical University of Munich, 85354 Freising, Germany; 5 Academic Rheumatology, Clinical Sciences Building, Nottingham City Hospital, University of Nottingham, Nottingham, UK

**Keywords:** microbiome, cardiometabolic diseases, obesity, phageome, bacteriophage, phage therapy, fecal virome transplant

## Abstract

Cardiometabolic diseases are leading causes of mortality in Western countries. Well-established risk factors include host genetics, lifestyle, diet, and the gut microbiome. Moreover, gut bacterial communities and their activities can be altered by bacteriophages (also known simply as phages), bacteria-infecting viruses, making these biological entities key regulators of human cardiometabolic health. The manipulation of bacterial populations by phages enables the possibility of using phages in the treatment of cardiometabolic diseases through phage therapy and fecal viral transplants. First, however, a deeper understanding of the role of the phageome in cardiometabolic diseases is required. In this review, we first introduce the phageome as a component of the gut microbiome and discuss fecal viral transplants and phage therapy in relation to cardiometabolic diseases. We then summarize the current state of phageome research in cardiometabolic diseases and propose how the phageome might indirectly influence cardiometabolic health through gut bacteria and their metabolites.

## Teaser

Kirk et al. explore the role of phages, bacteria-infecting viruses, in cardiometabolic health and highlight the therapeutic potential of fecal viral transplants and phage therapy as a strategy to modify gut bacteria and their metabolites and thus affect clinical outcomes.

## Introduction

In the last decades, there has been a dramatic worldwide increase in obesity and cardiometabolic diseases (CMDs) including type 2 diabetes mellitus (T2D), hypertension, cardiovascular disease (CVD), and nonalcoholic fatty liver disease (NAFLD).^1^ CMDs are multifactorial disorders, and traditional risk factors include environmental exposures, diet, lifestyle, and genetic and epigenetic factors.^1^ Recently, the gut microbiome has also emerged as a crucial player in CMDs, influencing various aspects of metabolic function and disease development.^2^^,^^3^ Indeed, gut bacteria regulate multiple host functions, including digestion, immunity, and endocrine function.^4^

Despite a huge research investment in the gut microbiome over the last decade, there are still some unanswered questions, conflicting results, and a paucity of gut microbiome-based therapies.^5^ Furthermore, gut microbiome research has been heavily biased toward the study of bacteria, when, in fact, viruses, archaea, and fungi are also present.^6^ These components both modulate bacterial populations and interact with human health directly.^6^ Specifically, bacteriophages (or simply, phages), bacteria-infecting viruses, are key drivers of bacterial community structure and function^7^ and have thus been associated with not only gastrointestinal diseases but also systemic health, including CMDs.^8^^,^^9^^,^^10^^,^^11^^,^^12^ By infecting gut bacteria, phages can (1) increase or decrease bacterial abundances^13^ and (2) alter the function of their bacterial hosts even if the population numbers of the host or the phage remain unchanged.^14^ This suggests that phages are indirectly associated with gut microbiome-associated diseases such as inflammatory bowel disease (IBD),^9^ irritable bowel syndrome,^8^ T2D^11^ and the metabolic syndrome (MetS).^10^

Because of their capacity to modulate bacterial composition and function in the gut microbiome, phages have been considered to be therapeutic options in disease states in which gut bacteria are known to play a role.^7^ Indeed, they have a narrow target host range, can remain active long after administration, and typically have minimal side effects or safety concerns for human hosts.^15^ However, a deeper understanding of their role in the gut microbiome and human health is required to enable therapeutic breakthroughs. Two therapies in which phages play a fundamental role are fecal virome transplantation (FVT), which has already been applied in T2D and obesity^16^^,^^17^^,^^18^ and phage therapy, which has been applied to various conditions, including gastrointestinal diseases, urinary tract infections, and antibiotic-resistant infections.^19^^,^^20^ However, the extent to which the phages are involved in CMDs and whether the aforementioned therapies represent viable options for their treatment are currently unknown.

In this review, we introduce the phageome as a component of the gut microbiome. We then provide an overview of its role in human health, with a focus on cardiometabolic health and the potential for FVT and phage therapy, with examples from clinical and preclinical models. We then discuss the current state of phage research in CMDs and the links with the bacterial component of the gut microbiome. A deeper understanding of the role of phages in CMDs through phageome research can lead to novel mechanistic understandings and therapeutic breakthroughs.

## The phageome as a component of the gut microbiome

### The gut microbiome and its components

**Figure 1:**
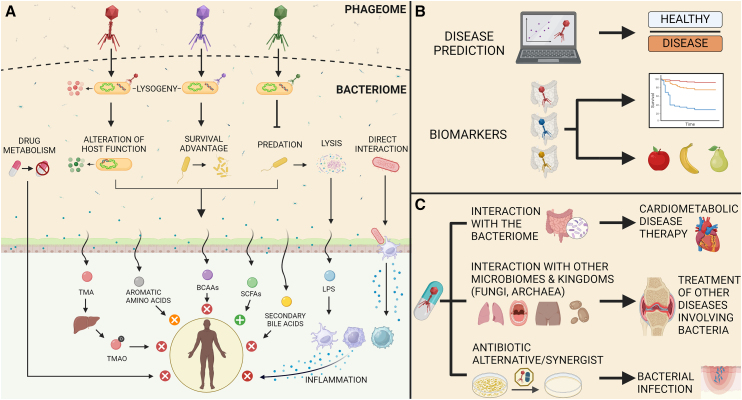
The gut phageome: Interactions, research applications, and therapeutic potentials (A) Interactions between bacteria, phages, microbially derived metabolites, and human health. (B) Applications of phages in research. (C) Public health applications and therapeutic potential of phages.

The gut microbiome refers to the collection of bacteria, viruses (including phages), fungi, archaea, and their genes that exist in the digestive tract. These biological entities coexist in harmony with the host, and perturbations of the gut microbiome can lead to negative health outcomes (Figure 1).^7^

Colonization of the gut microbiome starts at birth.^21^ Then, different factors shape its composition, including breastfeeding, use of antibiotics and medications, diet, and environmental exposures. Importantly, however, although the early-life influence is particularly strong, the gut microbiome reacts to changes at any point in the lifespan.^21^ This plasticity combined with the relationship with health outcomes has made the gut microbiome the center of much research over the last decade.^22^

Most studies so far have focused on the bacterial component of the gut microbiome, partly because 16S rRNA gene amplicon sequencing has enabled the precise and sensitive detection of bacteria due to its ubiquity within the kingdom.^23^ However, technological advances such as whole metagenome shotgun sequencing are also able to incorporate nonbacterial organisms in their analyses, including viral species.^24^

### The phageome

The community of viruses that reside in the gut is mainly composed of phages (the phageome). Phage density increases along the gastrointestinal tract and reaches a maximum of 10^8^–8^10^ phage virions per gram of feces in the large intestine.^25^ Despite their abundance and potential roles in shaping gut microbiome composition and function,^25^ phages remain largely uncharacterized, with up to 90% of all viral sequences in databases unknown (the “viral dark matter”).

**Figure 2:**
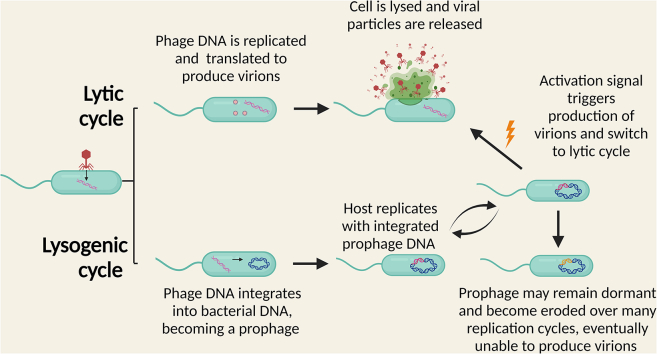
Life cycles of phages

Phages can be broadly categorized based on their lifestyles, namely lysis and lysogeny^26^ (Figure 2). Virulent phages have a lytic life cycle in which infection of their bacterial hosts is followed by DNA replication and lysis of the bacterium, causing the release of newly synthesized virions.^26^ This affects bacterial production in the gut and can shift gut bacterial composition.^27^ Phages are crucial in maintaining high prokaryotic richness in one environment by preferentially targeting the most abundant species of bacteria (the “kill-the-winner” hypothesis^28^). Conversely, in the gut, where microbial abundance and growth rates are high, phages may prefer to adopt temperate behavior and enter the lysogenic lifestyle (“piggyback-the-winner”).

Lysogeny involves integrating the genetic material of the phage into the bacterial genome,^26^ thus becoming a prophage that is automatically replicated and maintained when the bacterial cell replicates.^26^ The prophage may stay inactive across many rounds of replication, usually requiring an activation signal within the host cell (e.g., stress) to become transcribed and translated, resulting in the release of newly formed virions.^25^ Although this can slow bacterial production since resources are directed away from the bacterium and toward virion synthesis,^26^ bacterial fitness is often increased in a process resembling symbiosis.^27^ Alternatively, prophage DNA may be altered due to accumulated mutations over many bacterial reproduction cycles, eventually losing its ability to be transcribed and produce virions.^25^

During their life cycles, phages have the capacity to transfer genes from one bacterial species to another (horizontal gene transfer).^27^^,^^28^^,^^29^ This is done via processes of transduction that can be either generalized (i.e., a random piece of the host DNA is incorporated during cell lysis) or specialized (i.e., a prophage imprecisely excises itself from a host genome and incorporates some of the flanking host DNAs into their own^29^). Transduction has the potential to modulate the fitness of the bacterial hosts of the phages.^27^^,^^28^^,^^29^ For instance, phage-encoded auxiliary metabolic genes (AMGs) originated from bacteria and then incorporated into the phage genome, are pervasively found in phages.^30^ AMGs can alter metabolic processes in the gut by reprogramming bacterial host metabolism and encoding bacterial exotoxins.^30^ As such, AMGs and phages encoding them have been associated with immunomodulation, lipopolysaccharides (LPSs), and intestinal permeability, all of which are associated with CMDs.^30^

### Phage bioinformatics

Historically, phageome characterization relied on cultivation-based methods, including isolating viral-like proteins (VLPs) from ecological environments and enriching host bacteria. These methods have led to the isolation of phages from certain model hosts,^31^ and a recent large-scale phage cultivation study has successfully isolated 209 phages for 42 commensal human gut bacterial species.^32^ However, although cultivation studies facilitate bacterial phage-host assignment and do not rely on reference databases, they remain limited in scale. They are also restricted to gut bacteria that can be cultured *in vitro*. Hence, bioinformatic methods are the most common and effective approaches for characterizing the gut phageome. Recent advances in metagenomic sequencing and computational tools for the analysis of data thereof have enabled a more in-depth analysis of the complexity and richness of phages in the gut microbiome.^14^^,^^33^^,^^34^

Metagenomic sequencing involves first the extraction of nucleic acid material from a sample (e.g., feces), which may be total DNA, only the viral fraction, or both.^35^ A library is then prepared from which the extracted genetic material is sequenced, followed by quality control steps on the raw reads.^24^ Reads remaining after quality control are then annotated by mapping reads to an existing bacteriophage database or by *de novo* assembly. Mapping reads to a reference database allows the instant identification of the species present in a sample; however, these approaches are limited to the taxonomic information available in the reference databases used.^24^ However, *de novo* assembly, being independent of reference databases, enables a much more complete picture of the phageome but is sensitive to the software used for assembly.^28^ Pipelines including MetaPhlAn4^34^ and ViroProfiler^36^ estimate phageome composition from shotgun metagenome data. The latest release of MetaPhlAn4^34^ includes over 162,000 viral sequences, whereas ViroProfiler^36^ is a containerized metagenomic data analysis tool with capabilities such as viral discovery, taxonomy assignment, functional annotation, and host and replication cycle predictions.

These technological advances have led to a better characterization of the phage component of the microbiome.^27^ Among the most common and well-studied phages in the gut, there are (1) the order Caudovirales, with double-stranded DNA (dsDNA) genomes and a tail structure,^28^ and (2) the *crAssphages*, so named due to the cross-assembly of phage sequencing data.^37^ *crAssphages* have unique genetic sequences that make up a significant portion (up to 90%^37^) of VLP-derived metagenomes in some populations, including Western, Korean, and Malawian.^37^^,^^38^ In addition, *crAssphages* bear no resemblance to any known phages and have been detected in more than 50% of the gut phageomes of the Western population,^39^ are associated with industrialization, and both positive and negative associations have been found with obesity across its various subfamilies.^40^^,^^41^

### Predicting bacterial hosts

Because phages modulate the gut bacteria composition, it is important to understand which bacteria they infect. Various approaches are involved in predicting bacterial hosts of phages.^42^ These include comparing genetic homology between phages and bacteria and investigating phage-host abundance profiles.

Homology between phage and bacterial genetic sequences can reveal previous infection events between a phage and a bacterial species and can arise due to horizontal transfer, prophage integration, insertion sites (e.g., tRNAs), or CRISPR spacer sequences.^42^^,^^43^ For example, CRISPR spacers have been used to identify the hosts of thousands of newly discovered phages from whole-community metagenomes in the NCBI Assembly database, with hosts including the *Bacteroides* genera, implicated in CMDs,^44^ and whose fitness was influenced by the phages infecting them.^45^ In contrast, phage-host abundance methods are based on the idea that the interaction dynamics of phages and bacteria can be used to assign bacterial hosts to phages.^46^ With data obtained from repeated sampling, these correlation-based approaches aim to assign hosts by analyzing common trends between phages and bacteria in an environment.^43^^,^^46^ In doing so, assumptions are made about the relationships between phages and bacteria, which, given the complex dynamics of the gut microbiome, may lead to bias.^43^^,^^46^^,^^47^

## Manipulating the gut microbiome: Phage therapy and FVT

FVT and phage therapy are two different approaches that involve the use of viruses and phages to modify gut microbiome composition and potentially gain therapeutic benefits. FVT involves the transfer of viral components from the stool of a healthy donor to the gut of a recipient to restore the microbiome.^48^ Phage therapy, conversely, uses a targeted approach to isolate and transplant phages that are effective against the specific bacterial strain causing the infection.^15^

### Fecal virome transplant

Unlike fecal matter transplantation (FMT), which transfers a wide range of microorganisms, FVT specifically targets the virome by transferring only viral components (including phages) from fecal matter. The fecal matter of the donor is first treated to remove intact bacterial cells via a size exclusion filter^49^ and then transplanted into the recipient with the aim of manipulating bacterial populations.^7^ Metabolites, macronutrients, and bacterial cell wall components will, however, remain in the fecal matrix.^50^ FVT has the advantage of reducing risks associated with FMTs such as bacterial infection.^7^

In preclinical studies, a reduction in weight and an improvement in oral glucose tolerance,^16^^,^^18^ liver pathology, adipose inflammation, and glucose clearance^51^ were observed in (high-fat diet-induced) obese mice receiving FVT from lean donor mice. Although FVTs have not yet been applied to CMD in humans, FMTs, which lead not only to the transplantation of bacteria but also of viruses and phages, have. Changes in the phageome following FMTs have been reported for various conditions, including *Clostridium difficile* infection, IBD, and autism spectrum disorder, among others.^52^ In a recent study, Manrique and colleagues transplanted fecal matter from five healthy donors to six individuals with MetS and observed a significant change in the gut phageomes of the recipients.^17^ Phageome richness and similarity between the phageome in the recipient post-transplantation and the donor phageome were correlated to FMT success.^17^ Although the sample size was small and it was not possible to assess whether phage community changes were a driving force in reshaping the gut microbiome post-transplant or simply a secondary reaction to bacterial changes, the study suggests that changes in the phageome correlate with clinical outcomes, including CMD.^17^

#### FVT challenges

Whereas FVTs pose potential advantages to FMTs due to the elimination of the bacterial components and thus the transmission of unwanted pathogens, the current lack of studies in this area prevents a deeper understanding of their effects, especially in the long term.^49^ In addition, unlike traditional medicines or antibiotics, phage-based treatments such as FVT involve the use of biological agents that could remain active in the gut microbiome of the recipient indefinitely.^15^^,^^53^ Although this could lead to long-term treatment success, co-occurring side effects due to the treatment may also persist.

Another significant challenge to the FVT is the transfer of unwanted viruses that are known to reside in the gut (e.g., herpesvirus) from the donor to the recipient with unknown consequences upon transmission.^54^ A thorough screening of the donor virome would therefore be needed before transplantation to characterize the viral species and the encoded genes.^54^ Alternatively, the filtrate would need to be modified before transplantation by using a solvent treatment to inactivate enveloped (eukaryotic) viruses,^51^ leaving most of the phage community intact. Still, even with eukaryotic viruses eliminated, much of the phageome remains uncharacterized,^55^ which motivates the need for a better understanding of the gut phageome in human health.

### Phage therapy

A key property of phages is their ability to target specific bacteria while leaving off-target species largely unaffected, which has led to their use as a treatment option known as phage therapy.^15^ Phage therapy involves using phages to target specific bacteria involved in disease progression.^15^ Recent research findings suggest the use of phages as an alternative to antibiotics due to their narrower target range and fewer off-target effects on other bacterial species, minimal side effects on human hosts, and their ability to coevolve with their bacterial hosts.^15^ These theoretical advantages of using phages have motivated efforts such as the Centre for Phage Research in Leicester, UK, which provides a biobank repository and plans to host a national library of phages to facilitate efforts in phage therapy.^56^

Although phage therapy has not yet been applied in CMDs, research on its application in other diseases can be informative. For instance, a recent systematic review of 27 studies and 165 patients supported the efficacy and safety of phage therapy in the treatment of infectious diseases caused by various multidrug-resistant strains of bacterial species.^57^ This included using phage therapy to target *Escherichia coli, Klebsiella pneumoniae*, and *Streptococcus*,^57^ all of which are known to be associated with several CMDs.^58^^,^^59^^,^^60^^,^^61^

Moreover, promising results were reported in the double-blinded crossover PHAGE study investigating the effect of a combination of phages targeting *E. coli* in participants with gastrointestinal complaints.^62^ Indeed, a significant reduction in *E. coli* populations was reported, whereas the non-target populations remained largely unchanged.^62^ In addition, preclinical studies consistently supported the safety of phage therapy and reported minimal effects on commensal bacteria.^15^

#### Phage therapy challenges

Because CMDs are usually characterized by a general dysbiosis and not by the dominance of a particular species,^63^ phage therapy in the treatment of CMDs may involve the development of a phage cocktail. Moreover, there are still challenges around the mode of delivery, the dosage, the stability of phage preparations (ensuring that the administered phages can reach their target location and infect their target host), and the ethical implications of using phage therapy as a treatment.^64^

## The role of phages in CMDs

To maximize the potential of phage-based therapies, a deeper understanding of how the phageome is altered in CMDs and how this relates to the bacterial component of the gut microbiome is required. Research in this area is limited but continuing to grow and is discussed in detail below.

### Gut bacteria

**Figure 3:**
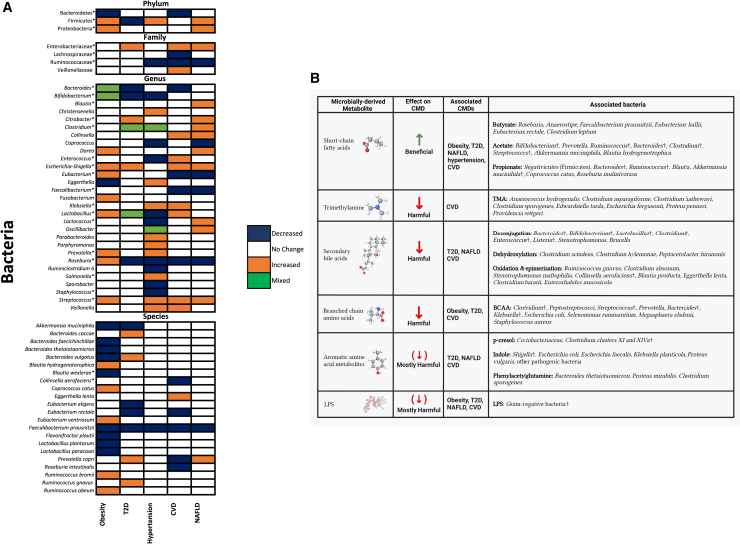
Gut bacteria, microbial metabolites, and CMD associations (A) Bacteria associated with CMDs by taxonomic rank. Orange indicates a positive association, blue indicates a negative association, white indicates a lack of association, and green indicates discordant results. The asterisk indicates that phages have been identified for the bacteria as reviewed here. (B) Major microbially derived metabolites, their associated bacteria, and their effect on cardiometabolic health. The cross symbol signifies bacteria for which phages have also been identified in the studies described in the text. Due to the variety of aromatic amino acid metabolites and their associated bacteria, only a few key examples are provided. Parentheses around an arrow convey that most of the compounds in a given class have the effect represented by the arrow, although some may also have the opposite effect, as is the case with certain aromatic amino acid metabolites and some LPS molecules.

Gut bacteria composition and diversity, as well as gut microbiome function, have been consistently implicated in CMDs, including obesity, T2D, hypertension, CVD, and NAFLD, among others, as summarized in Figure 3A and Table S1. For instance, a reduction in gut microbiome diversity and *Ruminococcaceae*, *Roseburia*, *Faecalibacterium Prausnitzii*, and *Akkermansia*, and an increase in *Enterobacteriaceae*, *Escherichia-Shigella*, *Klebsiella*, *Lactobacillus*, and *Streptococcus* have been reported in individuals with CMDs^4^ (Figure 3A). There are several mechanisms whereby the gut microbiome influences human health, including affecting gut permeability and thus regulating excretion and absorption; affecting inflammation through the activation of immune cells and the production of proinflammatory and anti-inflammatory signaling molecules; affecting neurotransmitter production and hormonal regulation; and, especially, by producing bacterial metabolites, including short-chain fatty acids, secondary bile acids, and branch chain amino acids. Bacterial metabolites are released into the bloodstream and are responsible for several metabolic conditions, including insulin resistance, diabetes, and obesity, thus mediating microbial effects on human health^65^ (see Figure 3B; Table S2).

Bacterial metabolites are indeed the key agents involved in the role of the gut microbiome in CMD. For instance, butyrate-producing bacteria are associated with a lower risk of T2D,^66^ acetate has been shown to mediate the effect of gut bacteria composition on visceral fat, and the gut bacteria-produced secondary bile acid isoursodeoxycholate is associated with liver function, postprandial lipemia, and inflammation.^67^

### Gut phageome

**Figure 4:**
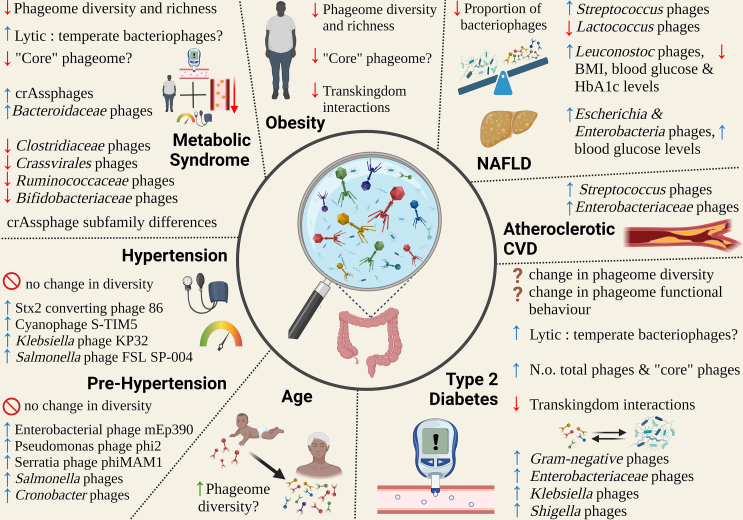
Association between the human phageome and CMDs

In contrast to the bacterial component of the gut, studies investigating the role of the gut phageome in CMDs are still in their infancy. Results are not always consistent across different disease states, and studies tend to be underpowered and have small sample sizes (see Figure 4; Table S3). Below, a summary of the existing studies by CMD is provided.

#### Obesity

A different phageome profile was observed in individuals with obesity compared to healthy controls or individuals with other CMDs.^11^^,^^60^^,^^68^ Differences in phages were also observed in 49 individuals before and after obesity intervention treatment (e.g., surgery, diet, exercise)^69^ and in obese children compared to normal-weight controls in *crAssphage* subfamilies^41^ (a taxonomic rank between the family and genus levels^70^). Moreover, a large study including 4,198 Japanese individuals reported a positive correlation between dsDNA phage diversity and BMI,^71^ whereas results were somewhat inconsistent in other smaller studies (n < 229).^11^^,^^68^^,^^69^

#### T2D

Individuals with T2D have been found to present differences in gut phageome composition compared to healthy controls.^11^^,^^72^^,^^73^^,^^74^ This includes a higher abundance of common phage operational taxonomic units (i.e., those present in over two-thirds of the sample),^73^ *Shigella* and *Xylella* phages^74^; *Enterobacteria phage cdtl*, *Enterobacteria phage ES18*, *Klebsiella phage KP34*, *Salmonella phage ST64T*^72^; *Cellulophaga phage* *and* *Bacteroides phage*^72^; lower levels of *Flavobacterium*, *Cellulophage*, *Staphylococcus*, *Synechoccus*, *Curvibacter*, *Clostridoides*, *Tenacibaculum*, *Paenibacillus*, *Lactobacillus*, *Listeria*, and *Citrobacter* phages^74^; *Brochothrix phage A9*, *Brochothrix phage NF5*, *Enterococcus phage phiFL2A*, and *Salmonella phage* PVP-SE1^72^; and *Thermoanaerobicbacterium phage*, *Verrucomicrobia phage*, and *Proteus phage*^11^; and alterations at the family level in individuals with T2D as compared to controls.^73^^,^^74^ In addition, the use of diabetes medication was found to correlate to dsDNA phageome composition.^71^ Chen and coworkers observed that certain phages, including *Bacillus*, *Enterococcus*, *Streptococcus*, and *Klebsiella*, correlated with fasting blood glucose and insulin, postprandial insulin, highly sensitive C-reactive protein, and free thyroxine.^72^ Furthermore, the gut phageome of individuals with T2D appeared more perturbed than the gut phageome of individuals with obesity when compared to healthy controls.^11^ All of the differences in phages observed in those with obesity were also observed in those with T2D but not vice versa. Phageome richness and diversity were reported to be lower in individuals with T2D,^74^ although results are inconsistent across studies.^11^^,^^71^^,^^72^^,^^73^

#### MetS

Studies report compositional alterations in individuals with MetS.^10^^,^^41^^,^^68^^,^^75^ For instance, a recent study in 196 Dutch individuals reported that individuals with MetS have a higher abundance of *Bacteroidaceae*- and *Streptococcaceae*-infecting phages and a lower abundance of *Bifidobacteriaceae*-infecting phages compared to healthy controls.^10^ In addition, a new phage family, called *Candidatus Heliusviridae*, was present in more than 96% of participants and had subfamilies that were related to MetS.^10^ The Crassvirales order was also significantly less prevalent in the MetS phageomes compared to controls.^10^ Individuals with MetS also have a different relative abundance of *crAssphage* compared to healthy controls, although the direction of the association is not clear.^41^^,^^68^^,^^75^

In contrast to the results found in T2D,^73^ highly prevalent phages appear to be reduced in individuals with MetS.^10^^,^^68^ De Jonge and coworkers identified two viral clusters present in >30% of controls, whereas these were not present in MetS, and there were no viral clusters present in >30% of the individuals in MetS.^10^ Similarly, Bikel et al. found that the average prevalence of highly abundant (present in >80%) phage contigs in the normal-weight group decreased from 91.54% to 76.35% in obesity and to 68.27% in MetS.^68^

A very small study reported an increase in richness and diversity in schoolchildren with obesity and MetS compared to healthy controls.^68^ In contrast, in a larger study in adults, lower richness and diversity were observed in those with MetS.^10^ Phage richness was also negatively correlated with obesity, blood glucose, blood pressure, and triglycerides.^10^

#### Hypertension

Han and colleagues found that dominant phages across levels of hypertension were different.^76^ For instance, the gut phageome of individuals with hypertension was reported to be dominated by *Klebsiella phage KP32*, *Cyanophage S-TIM5*, and *Salmonella phage FSL SP-004*; that of individuals with prehypertension by *Cronobacter phage CR3*, *Cronobacter phage ENT39118*, and *Cronobacter phage phiES15*; and finally, *Salmonella phage vB-SemP-Emek*, *Pseudomonas phage PaMx11*, and *Gordonia phage GTE8* dominated the gut phageome of controls.^76^ No difference in diversity was observed across the groups. In addition, the dsDNA phageomes of 4,198 Japanese individuals were associated with hypertension.^71^ However, due to the limited number of studies, more research is required to understand the role of the phageome in hypertension, including research investigating the relation of the phageome to blood pressure as a continuous outcome.

#### NAFLD

Only one study investigated the phageome in NAFLD,^12^ in which a reduction in *Lactococcus* and an increase in *Streptococcus* phages in VLP fractions in NAFLD patients with higher NAFLD activity scores (NAS; a measure of NAFLD severity based on histological lesions of the liver^77^) was reported.^12^ Moreover, VLPs from patients with NAS of 5–8 had lower phageome diversity and a lower proportion of phage to nonphage genetic material compared with those with NAS 0–4 or controls.^12^ More studies investigating phageome alterations in NAFLD are required to validate these results.

#### CVD and hypertriglyceridemia

Jie and colleagues reported that individuals with atherosclerotic CVD have a different phageome profile compared to healthy controls with an enrichment in *Enterobacteriaceae-* and *Streptococcus-*infecting phages.^60^ They observed that individuals with atherosclerotic CVD had a phageome profile similar to that of individuals with cirrhosis, whereas little overlap was observed with those suffering from obesity, T2D, and rheumatoid arthritis.^60^ The dsDNA phageome was also found to correlate with CMD medications, including platelet aggregation inhibitors and statins.^71^

## Potential mechanisms of linking the phageome with CMD

Recent studies investigated the gut bacteria–phage relationship and reported alterations in phages infecting bacteria related to CMDs, including *Bacteroides*,^10^^,^^11^^,^^73^^,^^74^ *Bifidobacterium*,^10^ *Blautia*,^10^ *Clostridium*,^10^^,^^73^ *Escherichia-Shigella*,^11^^,^^69^^,^^72^^,^^73^^,^^74^ *Lactobacillus*,^11^^,^^73^^,^^74^ *Klebsiella*,^76^ *Roseburia*,^10^ and *Streptococcus*^11^^,^^12^^,^^60^^,^^72^ (see Figure 3A).

In a study of 90 individuals with T2D and 42 healthy controls, Fan and colleagues identified several significant associations between phages and both short-chain fatty acid (SCFA)-producing bacteria (e.g., *F. prausnitzii, Roseburia faecis, R. inulinivorans*) and other bacteria with known relationships to CMDs (including *Akkermansia muciniphila*).^74^ Some of the identified associations appeared to be disease-specific, because when stratifying by disease status, the phage–bacteria correlations were not always consistent. For instance, *Shigella* phage correlated with the genera *Bluatia*, *Bacteroides*, and *Clostridium* only in those with T2D, whereas *Pseudomonas phage* was found to correlate with *A. muciniphila* and *Ruminococcus bromii* only in controls.^74^ The results suggest that transkingdom interactions are altered in T2D and shed light on how changes in gut bacteria co-occur with changes in gut phages.

Another study in T2D used network analysis to show that the bacterial genera with the most connections to phages were *Escherichia* and *Bacteroides*, two genera that are commonly associated with CMDs^73^ (Figure 3A). Consistently, other studies report changes in phage–bacteria interactions, including a reduction in the number of correlations between phages and bacteria in obesity, T2D, and hypertension.^72^^,^^76^ Besides influencing bacterial population numbers, phages can provide accessory genes via prophage integration and horizontal gene transfer, therefore influencing the metabolic activity of their hosts.^10^^,^^73^

A switch toward increased lytic phages or their activity has been suggested in T2D and MetS.^10^^,^^72^^,^^73^ For example, Chen and colleagues sampled the extracellular phageome using VLPs in 17 diabetic patients and 29 nondiabetic controls and found a positive correlation between Gram-negative phages and their bacterial hosts, especially for *Enterobacteriaceae* and phages of members of this family, such as *Escherichia*, *Salmonella*, *Enterobacter*, *Shigella*, *Klebsiella*, and *Enterobacteria* phages.^72^ It is thought that an elevation in Gram-negative bacteria and their phages causes a “lytic switch,” leading to the release of bacterial cell components and inflammation, thus contributing to metabolic disease pathology.^72^^,^^78^^,^^79^^,^^80^ LPS was also elevated in the study of Chen and colleagues.^72^ In further support of this, Ma et al. also report a positive relationship between *Enterobacteria* and *Escherichia* and their phages, which is elevated in T2D, and de Jonge and colleagues found lower intracellular phage-to-bacterial ratios and higher viral counts in the VLP fraction, both of which suggest a lower lysogenic phage behavior.^10^^,^^73^ However, the results are somewhat in contrast to those of Fan and colleagues, who found an increased number of positive phage–bacterial correlations and a decreased number of negative ones in T2D, which would not be expected under increased lysis.^74^ Taken together, it is currently unclear whether a lytic shift occurs in CMDs, although this could be an interesting hypothesis to explore in future research.

In conclusion, the available studies, albeit limited, show a role for the phageome in CMDs and support a role for phage-based therapies in the manipulation of the bacterial component of the gut microbiome. In phage therapy, this would involve engineering phages to be delivered to the gut, where they would modify the abundance of CMD-associated bacteria (e.g., by increasing the abundance of SCFA-producing bacteria) or modify their metabolic behavior. Due to the variability of microbial alterations across different CMDs (see Figure 3A), this will probably be done on a disease- or even case-specific basis, which increases the technical challenges and costs. In FVT, this would involve the identification of healthy donor phageomes to be used to restore CMD-associated bacterial dysbiosis. However, the potential irreversible transfer of undesirable viruses and the consequences thereof must be carefully considered if FVTs are to be used in the treatment of CMDs.

## Conclusion and future perspectives

The emerging role of the phageome in CMDs gives rise to the possibility of using phages for diagnosis and treatment. The associations between phages and CMDs and other diseases suggest that phages represent a potential biomarker reservoir that may improve disease prediction and prognosis compared to traditional biomarkers or bacterial data alone. In terms of treatment, key properties of phages such as host specificity and minimal off-target effects^15^ make them attractive options for altering the bacterial component of the gut microbiome, which is implicated in CMDs.^2^ Although phage-based therapies and FVT are in their infancy, and randomized controlled trials (RCTs) supporting their use are limited,^17^ their translational potential presents a promising avenue for addressing the pressing challenges in the realm of CMD treatment.

Technological advancements and modern bioinformatics techniques are leading to a better characterization of the gut phageome and its role in CMDs, and future work will certainly benefit from the rapidly evolving field of artificial intelligence, which will have applications such as improving the quality of metagenomic samples, annotating phages from these samples, and predicting bacterial hosts of the phages that are identified. To fully capitalize on these advantages, however, more work in larger samples is needed to improve the statistical power of tests of association between phages and bacteria or disease outcomes. In addition, RCTs and longitudinal evidence are needed to bridge the gap between basic research and clinical applications, and interactions across the diverse array of taxonomic kingdoms in the gut (archaea, bacteria, fungi) should be considered to provide a more complete picture of the gut microbiome landscape. Exclusively studying bacteria may fail to account for the role of phages in diseases with a nonbacterial etiology.

Moreover, there has been much research interest in using phage therapies for drug-resistant bacterial infection, particularly in the context of antibiotic resistance.^15^^,^^81^ Because phages are highly specific, they leave off-target species, including beneficial bacteria, unaffected.^15^ They may also work synergistically with traditional antibiotics, enhancing their efficacy.^81^

Finally, the advantages of phage therapies and FVT can be extended beyond bacterial infections to other diseases in which bacteria are involved, to other organ microbiomes (oral, skin, lung, and vaginal). Examples may include endometriosis^82^^,^^83^ and the links between periodontitis and rheumatoid arthritis,^84^^,^^85^ where oral and vaginal microbiome features are involved in symptoms.

## Supplemental information

Document S1. Tables S1–S3

Document S2. Article plus supplemental information
