# Supplementary material for: Bacteriophages, gut bacteria, and microbial pathways interplay in cardiometabolic health
Source: Cell Rep. 2024 Jan 31;43(2):113728. doi: 10.1016/j.celrep.2024.113728 (PMC11554570; doi:10.1016/j.celrep.2024.113728)
Supplement: Document S1. Tables S1–S3 [file mmc1.pdf]

Cell Reports, Volume *43*

Supplemental information

Bacteriophages, gut bacteria, and microbial pathways interplay in cardiometabolic health

Daniel Kirk, Ricardo Costeira, Alessia Visconti, Mohammadali Khan Mirzaei, Li Deng, Ana M. Valdes, and Cristina Menni

Supplementary Material

Contents

Table S1: Bacteria at different taxonomic ranks and their alterations across various cardiometabolic diseases, with corresponding references.

Table S2: Microbially derived metabolites, bacteria associated with their production, and their association with cardiometabolic diseases.

Table S3: Association between the human gut phageome and CMD.

Supplementary References

Table S1: [Bacteria at different taxonomic ranks and their alterations across various cardiometabolic diseases, with corresponding references]. Related to Figure 3A.

Bacteria	Obesity	Type 2 Diabetes	Hypertension	CVD	NAFLD
**Phylum**					
Bacteroidetes	↓[S1,S2]			↓[S3]	
Firmicutes	↑[S2]	↓[S4,S5]	↑[S6]		↑[S7]
Proteobacteria	↑[S2]				↑[S8]
**Family**					
Enterobacteriaceae		↑[S9]		↑[S10,S11]	↑[S8]
Lachnospiraceae				↓[S11,S12]	
Ruminococcaceae			↓[S13]	↓[S11,S12,S14]	↓[S15]
Veillonellaceae				↑[S16]	
**Genera**					
Bacteroides	↑↓[S17]	↓[S18,S19]		↓[S10]	
Bifidobacterium	↑↓[S2,S17]	↓[S19]	↓[S20]		
Blautia					↑[S21]
Christensenella			↑[S22]		
Citrobacter		↑[S9]			↑[S9]
Clostridium		↑↓ [S5,S23,S24]	↑↓[S22,S25]		↑[S9,S21]
Collinsella				↑[S16,S26]	↑[S9,S15,S27]
Coprococcus			↓[S20]		↓[S8,S21]
Dorea	↑[S17]				↑[S7,S8,S28]
Enterococcus			↓[S22]	↑[S16,S29]	
Escherichia-Shigella	↑[S17]	↑[S9,S30]		↑[S10,S29]	↑[S8,S9,S21]
Eubacterium	↑[S17]			↓[S26]	↓[S8]
Eggerthella	↓[S17]		↑[S25]		

Faecalibacterium				↓ [S10,S16,S29]	↓ [S8,S21,S27,S31]
Fusobacterium	↑ [S1,S17]				
Klebsiella			↑ [S13,S20,S25,S32]	↑ [S10,S12]	
Lactobacillus	↑ [S1,S2]	↑↓ [S19]	↓ [S6,S13,S22]	↑ [S3,S16]	
Lactococcus			↓ [S22]		↑ [S31]
Oscillibacter			↑↓ [S20,S22,S33]		↑ [S9]
Parabacteroides			↑ [S13,S22,S25]		
Porphyromonas			↑ [S20]		
Prevotella	↑ [S17]		↑ [S13,S20]		
Roseburia	↑ [S17]	↓ [S5,S19,S23]	↓ [S20,S25,S32]	↓ [S10,S26,S29]	↓ [S7,S21]
Ruminiclostridium 6			↓ [S34]		
Salmonella			↑ [S25]		
Sporobacter			↓ [S33]		
Staphylococcus			↓ [S22]		
Streptococcus	↑ [S17]		↑ [S25,S32]	↑ [S10]	↑ [S21,S27,S31]
Veillonella			↑ [S33]	↑ [S11,S12]	
**Species**					
Akkermansia muciniphila	↑ [S1,S2]	↓ [S19]			
Bacteroides caccae		↑ [S23]			
Bacteroides faecichinchillae	↓ [S35]				
Bacteroides thetaiotaomicron	↓ [S35]				
Bacteroides vulgatus	↓ [S2]	↑ [S36]			
Blautia hydrogenotorophica	↑ [S35]				
Blautia wexlerae	↓ [S35]				
Collinsella aerofaciens				↓ [S14]	
Coprococcus catus	↑ [S35]				
Eggerthella lenta				↑ [S10]	
Eubacterium eligens		↓ [S5]			

Eubacterium rectale		↓[S23]		↓[S29]	
Eubacterium ventriosum	↑[S35]				
Faecalibacterium prausnitzii	↓[S1]	↓[S5,S19,S23]	↓[S20,S25]	↓[S10]	↓[S31]
Flavonifractor plautii	↓[S35]				
Lactobacillus plantarum	↓[S1]				
Lactobacillus paracasei	↓[S1]				
Prevotella copri		↑[S28]		↓[S10]	↑[S27]
Roseburia intestinalis				↓[S10]	
Ruminococcus bromii	↑[S35]				
Ruminococcus gnavus		↑[S28]			
Ruminococcus obeum	↑[S35]				

Table S2: **[Microbially derived metabolites, bacteria associated with their production, and their association with cardiometabolic diseases].** Related to Figure 3B.

**Microbially-derived metabolite**	**Effect on CMD**	**Associated CMDs**	**Associated Bacteria**	**References**
Short-chain fatty acids	Beneficial	Obesity, T2D, NAFLD, hypertension, CVD	**Butyrate**: *Roseburia, Anaerostipe, Faecalibacterium prausnitzii, Eubacterium hallii, Eubacterium rectale, Clostridium leptum*<br><br>**Acetate**: *Bifidobacterium †, Prevotella, Ruminococcus †, Bacteroides †, Clostridium †, Streptococcus †, Akkermansia muciniphila, Blautia hydrogenotrophica*<br><br>**Propionate**: Negativicutes (Firmicutes), *Bacteroides †, Ruminococcus †, Blautia, Akkermansia mucinihila †, Coprococcus catus, Roseburia inulinivorans*	[S37–S39]
Trimethylamine	Harmful	CVD	**TMA**: *Anaerococcus hydrogenalis, Clostridium asparagiforme, Clostridium hathewayi, Clostridium sporogenes, Edwardsiella tarda, Escherichia fergusonii, Proteus penneri, Providencia rettgeri*	[S40]
Secondary bile acids	Harmful	T2D, NAFLD CVD	**Deconjugation**: *Bacteroides †, Bifidobacterium †, Lactobacillus †, Clostridium †, Enterococcus †, Listeria †, Stenotrophomonas, Brucella*<br><br>**Dehydroxylation**: *Clostridium scindens, Clostridium*	[S41]

			hylemonae, Peptacetobacter hiranonis<br><br>**Oxidation & epimerization**: *Ruminococcus gnavus, Clostridium absonum, Stenotrophomonas maltophilia, Collinsella aerofaciens †, Blautia producta, Eggerthella lenta, Clostridium baratii, Enterorhabdus mucosicola*	
Branched chain amino acids	Harmful	Obesity, T2D, CVD	**BCAA**: *Clostridium †, Peptostreptococci, Streptococcus †, Prevotella, Bacteroides †, Klebsiella †, Escherichia coli, Selenomonas ruminantium, Megasphaera elsdenii, Staphylococcus aureus*	[S42]
Aromatic amino acids	Mostly harmful	T2D, NAFLD CVD	**p-cresol**: *Coriobacteriaceae, Clostridium clusters XI and XIVa †*<br><br>**Indole**: *Shigella †, Escherichia coli, Escherichia faecalis, Klebsiella planticola, Proteus vulgaris, other pathogenic bacteria*<br><br>**Phenylacetylglutamine**: *Bacteroides thetaiotaomicron, Proteus mirabilis, Clostridium sporogenes*	[S43,S44]
Lipopolysaccharides	Mostly harmful	Obesity, T2D, NAFLD, CVD	**LPS**: Gram-negative bacteria†	[S45,S46]

Table S3: **[Association between the human gut phageome and CMD].** Related to Figure 4**.** The equals symbol (=) means no change; brackets around an arrow means low significance. Abbreviations: ACVD, atherosclerotic cardiovascular disease; CRP, C-reactive protein; IBS, irritable bowel syndrome; MetS, metabolic syndrome; RCT, randomised controlled trial; T2D, type 2 diabetes.

**Metabolic Disease**	**Subjects**	**Study Design**	**Techniques**	**Phageome Diversity**	**Key Findings**	**Author, Date**
Obesity, MetS	School Children	**Case-Control**<br><br>10 Obesity<br><br>8 MetS<br><br>10 Normal-weight	VLP	(↑)	Significantly different contigs between each group<br><br>↓ highly prevalent (>80%) phage contigs in the normo-weight group were reduced in disease states, going from an average prevalence of 91.54% (normo-weight) to 76.35% (obesity) to 68.27% (MetS)<br><br>Several disease-specific contigs correlate with bacterial taxa and bacterial abundances<br><br>Four highly abundant (present in 80% of all samples) contigs correlated with bacteria: *Collinsella aerofaciens, Parabacteroides distasoni, Phascolarctobacterium sp., Erysipelotrichaceae* family<br><br>Several highly abundant phage contigs correlated with BMI, waist circumference, glucose, triglycerides, and HDL, LDL, and total cholesterol	[S47]
Obesity, MetS	School Children	**Case-Control**	VLP	↓ (only crAssphage was assessed)	↓ prevalence, relative abundance, of crAssphage Alpha sub-family in MetS	[S48]

		10 Obesity<br><br>8 MetS<br><br>10 Normal-weight			↑ relative abundance of crAssphage Beta sub-family in MetS<br><br>↓ in putative crAssphage hosts Bacteroidetes, Bacteroidia, and Bacteroidales<br><br>↑ in putative crAssphage host *Collinsella*<br><br>Negative association between *Bacili* and crAssphage abundance	
MetS	Adults	**Case-Control**<br><br>97 MetS<br><br>99 Controls	WGS and VLP	↓	↓intracellular phage:bacteria ratios (↓lysogenic phages, ↑ lytic phages?)<br><br>↓*Crassvirales* phage prevalence in MetS<br>↑phages infecting *Streptococcaceae* (*Streptococcus salivarius*, *Streptococcus sp. HMSC078H03*) and *Bacteroidaceae* (*Bacteroides sp. 3_1_40A, Bacteroides sp. 2_1_16*) in MetS<br>↓phages infecting *Bifidobacteriaceae* (*Bifidobacterium longum, Bifidobacterium adolescentis, Bifidobacterium brevum*) in MetS<br><br>Prophages confer functions and alter metabolism of bacterial hosts<br><br>↑ phage richness correlates with ↓ obesity, blood glucose, blood pressure, and triglycerides<br><br>Phageome changes correlate with bacterial population changes	[S49]
MetS	Adults	**Case-Control**<br><br>30 MetS	qPCR	N/A	↑ Relative abundance of crAssphage in MetS	[S50]

		30 Control				
Obesity, T2D	Adults	**Case-Control**<br><br>128 Obese (including 74 T2D)<br><br>101 Controls	VLP	↓ (Hong Kong cohort)<br><br>= (Kunming cohort)	↓gut phage-bacterial transkingdom correlations in obesity versus lean controls<br><br>Differentially abundant phages in each group identified, including ↑ *Cellulophaga phage* and *Bacteroides phage* and ↓ *Thermoanaerobicbacterium phage*, *Verrucomicrobia phage* and *Proteus phage* in obese with T2D versus lean control<br><br>Phageome was more severely perturbed in obese with T2D versus obese without T2D<br><br>↓ positive correlations between *Enterobacteria* and *Pseudomonas* phages and healthy-associated bacteria, such as *Faecalibacterium prausnitzii* and *Roseburia intestinalis* in obesity and T2D.	[S51]
T2D	Adults	**Case-Control**<br><br>148 T2D<br><br>290 Non-T2D	WGS	N/A	= proportion of phages genes between T2D, healthy controls, and the other diseases studied (liver cirrhosis, IBS, Crohn's disease, colorectal cancer)<br><br>↓phage-bacteria interactions in T2D<br><br>= in phage function enrichment between disease and healthy<br><br>Differentially abundant phages between healthy versus any of the disease states No differentially abundant phages between each of the disease states	[S52]
T2D	Adults	**Case-Control**	VLP	=	= in abundance of phage families between T2D and healthy control	[S53]

		17 T2D<br>29 Controls			Differentially abundant phages in each group identified, including ↑ *Enterobacteria phage cdtl, Enterobacteria phage ES18, Klebsiella phage KP34,* and *Salmonella phage ST64T*, and ↓in *Brochothrix phage A9*, *Brochothrix phage NF5, Enterococcus phage phiFL2A,* and *Salmonella phage* PVP-SE1 in T2D.<br>↑relative abundance of gram-negative phages in T2D<br>↑*Enterobacteriaceae* and their phages in T2D<br>Correlations between phages and T2D clinical markers (fasting blood glucose and insulin, insulin 0.5 h and 2 h after a meal, highly sensitive CRP, and free thyroxine)	
T2D	Adults	**Case-Control**<br>71 T2D<br>74 Controls	WGS and VLP	=	↑number of phages in T2D<br>↑relative abundance of core (present in >66.6% of the samples) phage OTUs in T2D<br>*Escherichia* and *Bacteroides* genera had the highest number of interactions with phages<br>Abundance of phages did not correlate with the abundances of their inferred bacterial hosts	[S54]
T2D	Adults	**Case-Control**<br>90 T2D (of which 49 had neuropathy)<br>42 Controls	VLP	↓	Differentially abundant viral taxa (6), families (12) and species (81), of which 78% were phages<br>↓ *Flavobacterium, Cellulophaga, Staphylococcus , Synechoccus , Curvibacter , Clostridoides , Tenacibaculum , Paenibacillus , Lactobacillus, Listeria,* and *Citrobacter* phages, amongst others, in T2D	[S55]

					↑*Shigella* and *Xylella* phages in T2D<br><br>Loss of diverse viral functions in T2D, including viral replication and integration and phage lysis of bacteria host<br><br>Viral and bacterial diversity were positively correlated in T2D but not in controls<br><br>Alterations in transkingdom interactions, including both loss and gain of phage-bacteria interactions in T2D<br><br>↑positive viral-bacteria correlations and ↓negative viral-bacteria correlations in T2D<br><br>Correlation between *Streptococcus satellite phage* and five bacteria, including *Collinsella aerofaciens,* and *Pseudomonas phage* and four bacteria, including *Ruminococcus bromii* and *Akkermansia muciniphila*, lost in T2D	
Hypertension & prehypertension	Adults	**Case-Control**<br><br>99 Hypertensives<br><br>56 Pre-hypertensives<br><br>41 Controls	WGS	=	Dominant phages were different across each group, including:<br><br>• ↑ *Klebsiella phage KP32, Cyanophage S-TIM5, and Salmonella phage FSL SP-004* in hypertension*;*<br>• ↑ *Cronobacter phage CR3, Cronobacter phage ENT39118,* and *Cronobacter phage phiES15* in pre-hypertension;<br>• ↑ *Salmonella phage vB-SemP-Emek, Pseudomonas phage PaMx11* and *Gordonia phage GTE8* in controls<br><br>Phage-bacterial interactions altered in hypertension	[S56]
NAFLD	Adults	**Case-Control**<br><br>73 NAFLD (29 NAS Score 0-4,	VLP	↓	↓ proportion of phages in advanced NAFLD	[S57]

		44 NAS score 5-8, 37 F0–F1 fibrosis, 36 F2–F4 fibrosis)<br><br>13 Mild primary biliary cholangitis<br><br>22 Controls			↑*Leuconostoc phages* were associated with a ↓BMI, lower blood glucose and HbA1c levels, and ↑*Escherichia* and *Enterobacteria* phages were associated with ↑blood glucose levels	
ACVD, T2D, obesity	Adults 40-80 years old	Case-Control<br><br>218 ACVD, 187 Controls<br><br>171 T2D, 174 Controls<br><br>72 Obesity, 79 Controls	WGS	N/A	↑Mycobacterium phages Astraea, Gizmo, Pleione, ScottMcG, Spud, MoMoMixon, Aeromonas phage, Brochothrix phage A9, Synechococcus phage S-CAM1 in both CVD and obesity<br><br>↑*Mycobacterium phage Gumball* and *Escherichia phage wV7* in both CVD and T2D<br><br>Known hosts for phages enriched in ACVD were mainly of the family *Enterobacteriaceae* and *Streptococcus* genus.	[S10]
T2D, hypertension, obesity, age	Adults	**Cohort**<br><br>4198 Total (including 642 T2D)	WGS	↑ (with age)	Phageome composition associated with age hypertension, diabetes, and medication to treat metabolic conditions.<br><br>dsDNA phageome profile diversity was associated with age and diabetes medication use but not hypertension or diabetes.	[S58]
MetS	Adult males	**RCT, longitudinal**	VLP	↓ in non-responders versus	Phages unique to MetS replaced by new phages	[S59]

		(faecal transplant)<br><br>6 recipients, 5 donors		responders after treatment	Differentially abundant phages in responders versus non-responders post-treatment<br><br>phages acquired via treatment correlate with percentage increase of glucose disposal rate	
Obesity	Adults	**Intervention** (various obesity interventions)<br><br>21 pre-treatment and 28 post-treatment	WGS	↓ pre- versus post-obesity treatment	↑*Uncultured crAssphage*, *Enterobacteria phage AA91-ss* and *Enterobacteria phage 88* pre-obesity treatment<br><br>↑ *Enterobacteria phages HK629, lambda and EP460, Escherichia phages pro 147* and *483,* and *Yersinia phage L14_C.*, amongst others post-obesity treatment	[S60]

Supplementary References

S1. Crovesy, L., Masterson, D., and Rosado, E.L. (2020). Profile of the gut microbiota of adults with obesity: a systematic review. Eur J Clin Nutr *74*, 1251–1262. 10.1038/S41430-020-0607-6.

S2. Rinninella, E., Raoul, P., Cintoni, M., Franceschi, F., Miggiano, G.A.D., Gasbarrini, A., and Mele, M.C. (2019). What is the Healthy Gut Microbiota Composition? A Changing Ecosystem across Age, Environment, Diet, and Diseases. Microorganisms 2019, Vol. 7, Page 14 *7*, 14. 10.3390/MICROORGANISMS7010014.

S3. Yamashita, T., Emoto, T., Sasaki, N., and Hirata, K.I. (2016). Gut Microbiota and Coronary Artery Disease. Int Heart J *57*, 663–671. 10.1536/IHJ.16-414.

S4. Larsen, N., Vogensen, F.K., Van Den Berg, F.W.J., Nielsen, D.S., Andreasen, A.S., Pedersen, B.K., Al-Soud, W.A., Sørensen, S.J., Hansen, L.H., and Jakobsen, M. (2010). Gut microbiota in human adults with type 2 diabetes differs from non-diabetic adults. PLoS One *5*. 10.1371/JOURNAL.PONE.0009085.

S5. Karlsson, F.H., Tremaroli, V., Nookaew, I., Bergström, G., Behre, C.J., Fagerberg, B., Nielsen, J., and Bäckhed, F. (2013). Gut metagenome in European women with normal, impaired and diabetic glucose control. Nature *498*, 99–103. 10.1038/NATURE12198.

S6. Palmu, J., Salosensaari, A., Havulinna, A.S., Cheng, S., Inouye, M., Jain, M., Salido, R.A., Sanders, K., Brennan, C., Humphrey, G.C., et al. (2020). Association between the gut microbiota and blood pressure in a population cohort of 6953 individuals. J Am Heart Assoc *9*, 16641. 10.1161/JAHA.120.016641.

S7. Raman, M., Ahmed, I., Gillevet, P.M., Probert, C.S., Ratcliffe, N.M., Smith, S., Greenwood, R., Sikaroodi, M., Lam, V., Crotty, P., et al. (2013). Fecal Microbiome and Volatile Organic Compound Metabolome in Obese Humans With Nonalcoholic Fatty Liver Disease. Clinical Gastroenterology and Hepatology *11*, 868-875.e3. 10.1016/J.CGH.2013.02.015.

S8. Vijay, A., and Valdes, A.M. (2021). Role of the gut microbiome in chronic diseases: a narrative review. European Journal of Clinical Nutrition 2021 76:4 *76*, 489–501. 10.1038/s41430-021-00991-6.

S9. Frost, F., Kacprowski, T., Rühlemann, M., Pietzner, M., Bang, C., Franke, A., Nauck, M., Völker, U., Völzke, H., Dörr, M., et al. (2021). Long-term instability of the intestinal microbiome is associated with metabolic liver disease, low microbiota diversity, diabetes mellitus and impaired exocrine pancreatic function. Gut *70*, 522–530. 10.1136/GUTJNL-2020-322753.

S10. Jie, Z., Xia, H., Zhong, S.L., Feng, Q., Li, S., Liang, S., Zhong, H., Liu, Z., Gao, Y., Zhao, H., et al. (2017). The gut microbiome in atherosclerotic cardiovascular disease. Nature Communications 2017 8:1 *8*, 1–12. 10.1038/s41467-017-00900-1.

S11. Zeng, X., Gao, X., Peng, Y., Wu, Q., Zhu, J., Tan, C., Xia, G., You, C., Xu, R., Pan, S., et al. (2019). Higher Risk of Stroke Is Correlated With Increased Opportunistic Pathogen Load and Reduced Levels of Butyrate-Producing Bacteria in the Gut. Front Cell Infect Microbiol *9*. 10.3389/FCIMB.2019.00004.

S12. Liu, H., Chen, X., Hu, X., Niu, H., Tian, R., Wang, H., Pang, H., Jiang, L., Qiu, B., Chen, X., et al. (2019). Alterations in the gut microbiome and metabolism with coronary artery disease severity. Microbiome *7*, 1–14. 10.1186/S40168-019-0683-9/FIGURES/4.

S13. Masenga, S.K., Hamooya, B., Hangoma, J., Hayumbu, V., Ertuglu, L.A., Ishimwe, J., Rahman, S., Saleem, M., Laffer, C.L., Elijovich, F., et al. (2022). Recent advances in modulation of

cardiovascular diseases by the gut microbiota. Journal of Human Hypertension 2022 36:11 *36*, 952–959. 10.1038/s41371-022-00698-6.

S14. Menni, C., Lin, C., Cecelja, M., Mangino, M., Matey-Hernandez, M.L., Keehn, L., Mohney, R.P., Steves, C.J., Spector, T.D., Kuo, C.F., et al. (2018). Gut microbial diversity is associated with lower arterial stiffness in women. Eur Heart J *39*, 2390a–2397a. 10.1093/EURHEARTJ/EHY226.

S15. Astbury, S., Atallah, E., Vijay, A., Aithal, G.P., Grove, J.I., and Valdes, A.M. (2020). Lower gut microbiome diversity and higher abundance of proinflammatory genus Collinsella are associated with biopsy-proven nonalcoholic steatohepatitis. Gut Microbes *11*, 569–580. 10.1080/19490976.2019.1681861/SUPPL_FILE/KGMI_A_1681861_SM8563.DOCX.

S16. Cui, X., Ye, L., Li, J., Jin, L., Wang, W., Li, S., Bao, M., Wu, S., Li, L., Geng, B., et al. (2018). Metagenomic and metabolomic analyses unveil dysbiosis of gut microbiota in chronic heart failure patients. Sci Rep *8*. 10.1038/S41598-017-18756-2.

S17. Pinart, M., Dötsch, A., Schlicht, K., Laudes, M., Bouwman, J., Forslund, S.K., Pischon, T., and Nimptsch, K. (2021). Gut Microbiome Composition in Obese and Non-Obese Persons: A Systematic Review and Meta-Analysis. Nutrients *14*. 10.3390/NU14010012.

S18. Durack, J., and Lynch, S. V. (2019). The gut microbiome: Relationships with disease and opportunities for therapy. Journal of Experimental Medicine *216*, 20–40. 10.1084/JEM.20180448.

S19. Gurung, M., Li, Z., You, H., Rodrigues, R., Jump, D.B., Morgun, A., and Shulzhenko, N. (2020). Role of gut microbiota in type 2 diabetes pathophysiology. EBioMedicine *51*, 102590. 10.1016/J.EBIOM.2019.11.051.

S20. Li, J., Zhao, F., Wang, Y., Chen, J., Tao, J., Tian, G., Wu, S., Liu, W., Cui, Q., Geng, B., et al. (2017). Gut microbiota dysbiosis contributes to the development of hypertension. Microbiome *5*, 1–19. 10.1186/S40168-016-0222-X/FIGURES/7.

S21. Sharpton, S.R., Ajmera, V., and Loomba, R. (2019). Emerging Role of the Gut Microbiome in Nonalcoholic Fatty Liver Disease: From Composition to Function. Clinical Gastroenterology and Hepatology *17*, 296–306. 10.1016/J.CGH.2018.08.065.

S22. Dan, X., Mushi, Z., Baili, W., Han, L., Enqi, W., Huanhu, Z., and Shuchun, L. (2019). Differential Analysis of Hypertension-Associated Intestinal Microbiota. Int J Med Sci *16*, 872–881. 10.7150/IJMS.29322.

S23. Qin, J., Li, Y., Cai, Z., Li, S., Zhu, J., Zhang, F., Liang, S., Zhang, W., Guan, Y., Shen, D., et al. (2012). A metagenome-wide association study of gut microbiota in type 2 diabetes. Nature 2012 490:7418 *490*, 55–60. 10.1038/nature11450.

S24. Wu, H., Tremaroli, V., Schmidt, C., Lundqvist, A., Olsson, L.M., Krämer, M., Gummesson, A., Perkins, R., Bergström, G., and Bäckhed, F. (2020). The Gut Microbiota in Prediabetes and Diabetes: A Population-Based Cross-Sectional Study. Cell Metab *32*, 379-390.e3. 10.1016/J.CMET.2020.06.011.

S25. Yan, Q., Gu, Y., Li, X., Yang, W., Jia, L., Chen, C., Han, X., Huang, Y., Zhao, L., Li, P., et al. (2017). Alterations of the Gut Microbiome in Hypertension. Front Cell Infect Microbiol *7*. 10.3389/FCIMB.2017.00381.

S26. Karlsson, F.H., Fåk, F., Nookaew, I., Tremaroli, V., Fagerberg, B., Petranovic, D., Bäckhed, F., and Nielsen, J. (2012). Symptomatic atherosclerosis is associated with an altered gut metagenome. Nature Communications 2012 3:1 *3*, 1–8. 10.1038/ncomms2266.

S27. Ruuskanen, M.O., Åberg, F., Männistö, V., Havulinna, A.S., Méric, G., Liu, Y., Loomba, R., Vázquez-Baeza, Y., Tripathi, A., Valsta, L.M., et al. (2021). Links between gut microbiome

composition and fatty liver disease in a large population sample. Gut Microbes *13*, 1–22. 10.1080/19490976.2021.1888673/SUPPL_FILE/KGMI_A_1888673_SM1172.ZIP.
S28. Ruuskanen, M.O., Erawijantari, P.P., Havulinna, A.S., Liu, Y., Méric, G., Tuomilehto, J., Inouye, M., Jousilahti, P., Salomaa, V., Jain, M., et al. (2022). Gut Microbiome Composition Is Predictive of Incident Type 2 Diabetes in a Population Cohort of 5,572 Finnish Adults. Diabetes Care *45*, 811–818. 10.2337/DC21-2358.
S29. Zhu, Q., Gao, R., Zhang, Y., Pan, D., Zhu, Y., Zhang, X., Yang, R., Jiang, R., Xu, Y., and Qin, H. (2018). Dysbiosis signatures of gut microbiota in coronary artery disease. Physiol Genomics *50*, 893–903. 10.1152/PHYSIOLGENOMICS.00070.2018/ASSET/IMAGES/LARGE/ZH70111843130007.JPEG.
S30. Hartstra, A. V., Bouter, K.E.C., Bäckhed, F., and Nieuwdorp, M. (2015). Insights Into the Role of the Microbiome in Obesity and Type 2 Diabetes. Diabetes Care *38*, 159–165. 10.2337/DC14-0769.
S31. Caussy, C., Tripathi, A., Humphrey, G., Bassirian, S., Singh, S., Faulkner, C., Bettencourt, R., Rizo, E., Richards, L., Xu, Z.Z., et al. (2019). A gut microbiome signature for cirrhosis due to nonalcoholic fatty liver disease. Nature Communications 2019 10:1 *10*, 1–9. 10.1038/s41467-019-09455-9.
S32. Verhaar, B.J.H., Collard, D., Prodan, A., Levels, J.H.M., Zwinderman, A.H., Backhed, F., Vogt, L., Peters, M.J.L., Muller, M., Nieuwdorp, M., et al. (2020). Associations between gut microbiota, faecal short-chain fatty acids, and blood pressure across ethnic groups: the HELIUS study. Eur Heart J *41*, 4259–4267. 10.1093/EURHEARTJ/EHAA704.
S33. Sun, S., Lulla, A., Sioda, M., Winglee, K., Wu, M.C., Jacobs, D.R., Shikany, J.M., Lloyd-Jones, D.M., Launer, L.J., Fodor, A.A., et al. (2019). Gut Microbiota Composition and Blood Pressure. Hypertension *73*, 998–1006. 10.1161/HYPERTENSIONAHA.118.12109.
S34. Louca, P., Nogal, A., Wells, P.M., Asnicar, F., Wolf, J., Steves, C.J., Spector, T.D., Segata, N., Berry, S.E., Valdes, A.M., et al. (2021). Gut microbiome diversity and composition is associated with hypertension in women. J Hypertens *39*, 1810–1816. 10.1097/HJH.0000000000002878.
S35. Castaner, O., Goday, A., Park, Y.M., Lee, S.H., Magkos, F., Shiow, S.A.T.E., and Schröder, H. (2018). The Gut Microbiome Profile in Obesity: A Systematic Review. Int J Endocrinol *2018*. 10.1155/2018/4095789.
S36. Pedersen, H.K., Gudmundsdottir, V., Nielsen, H.B., Hyotylainen, T., Nielsen, T., Jensen, B.A.H., Forslund, K., Hildebrand, F., Prifti, E., Falony, G., et al. (2016). Human gut microbes impact host serum metabolome and insulin sensitivity. Nature 2016 535:7612 *535*, 376–381. 10.1038/nature18646.
S37. Venegas, D.P., De La Fuente, M.K., Landskron, G., González, M.J., Quera, R., Dijkstra, G., Harmsen, H.J.M., Faber, K.N., and Hermoso, M.A. (2019). Short Chain Fatty Acids (SCFAs)-Mediated Gut Epithelial and Immune Regulation and Its Relevance for Inflammatory Bowel Diseases. Front Immunol *10*. 10.3389/FIMMU.2019.00277.
S38. Nogal, A., Valdes, A.M., and Menni, C. (2021). The role of short-chain fatty acids in the interplay between gut microbiota and diet in cardio-metabolic health. Gut Microbes *13*, 1–24. 10.1080/19490976.2021.1897212.
S39. Reichardt, N., Duncan, S.H., Young, P., Belenguer, A., McWilliam Leitch, C., Scott, K.P., Flint, H.J., and Louis, P. (2014). Phylogenetic distribution of three pathways for propionate

production within the human gut microbiota. The ISME Journal 2014 8:6 *8*, 1323–1335. 10.1038/ismej.2014.14.

S40. Liu, Y., and Dai, M. (2020). Trimethylamine N-Oxide Generated by the Gut Microbiota Is Associated with Vascular Inflammation: New Insights into Atherosclerosis. Mediators Inflamm *2020*. 10.1155/2020/4634172.

S41. Guzior, D. V., and Quinn, R.A. (2021). Review: microbial transformations of human bile acids. Microbiome *9*, 1–13. 10.1186/S40168-021-01101-1/FIGURES/5.

S42. Gojda, J., and Cahova, M. (2021). Gut Microbiota as the Link between Elevated BCAA Serum Levels and Insulin Resistance. Biomolecules 2021, Vol. 11, Page 1414 *11*, 1414. 10.3390/BIOM11101414.

S43. Saito, Y., Sato, T., Nomoto, K., and Tsuji, H. (2018). Identification of phenol- and p-cresol-producing intestinal bacteria by using media supplemented with tyrosine and its metabolites. FEMS Microbiol Ecol *94*, 125. 10.1093/FEMSEC/FIY125.

S44. Lee, J.H., and Lee, J. (2010). Indole as an intercellular signal in microbial communities. FEMS Microbiol Rev *34*, 426–444. 10.1111/J.1574-6976.2009.00204.X.

S45. Steimle, A., Autenrieth, I.B., and Frick, J.S. (2016). Structure and function: Lipid A modifications in commensals and pathogens. International Journal of Medical Microbiology *306*, 290–301. 10.1016/J.IJMM.2016.03.001.

S46. Di Lorenzo, F., De Castro, C., Silipo, A., and Molinaro, A. (2019). Lipopolysaccharide structures of Gram-negative populations in the gut microbiota and effects on host interactions. FEMS Microbiol Rev *43*, 257–272. 10.1093/FEMSRE/FUZ002.

S47. Bikel, S., López-Leal, G., Cornejo-Granados, F., Gallardo-Becerra, L., García-López, R., Sánchez, F., Equihua-Medina, E., Ochoa-Romo, J.P., López-Contreras, B.E., Canizales-Quinteros, S., et al. (2021). Gut dsDNA virome shows diversity and richness alterations associated with childhood obesity and metabolic syndrome. iScience *24*. 10.1016/J.ISCI.2021.102900.

S48. Cervantes-Echeverría, M., Gallardo-Becerra, L., Cornejo-Granados, F., and Ochoa-Leyva, A. (2023). The Two-Faced Role of crAssphage Subfamilies in Obesity and Metabolic Syndrome: Between Good and Evil. Genes (Basel) *14*, 139. 10.3390/GENES14010139/S1.

S49. de Jonge, P.A., Wortelboer, K., Scheithauer, T.P.M., van den Born, B.J.H., Zwinderman, A.H., Nobrega, F.L., Dutilh, B.E., Nieuwdorp, M., and Herrema, H. (2022). Gut virome profiling identifies a widespread bacteriophage family associated with metabolic syndrome. Nat Commun *13*. 10.1038/S41467-022-31390-5.

S50. Bannazadeh Baghi, H., Naghili, B., Shanehbandi, D., and Ebrahimzadeh Leylabadlo, H. (2022). Evaluation of a human gut-associated phage and gut dominant microbial phyla in the metabolic syndrome. Clin Nutr ESPEN *50*, 133–137. 10.1016/J.CLNESP.2022.06.009.

S51. Yang, K., Niu, J., Zuo, T., Sun, Y., Xu, Z., Tang, W., Liu, Q., Zhang, J., Ng, E.K.W., Wong, S.K.H., et al. (2021). Alterations in the Gut Virome in Obesity and Type 2 Diabetes Mellitus. Gastroenterology *161*, 1257-1269.e13. 10.1053/J.GASTRO.2021.06.056.

S52. Li, M., Wang, C., Guo, Q., Xu, C., Xie, Z., Tan, J., Wu, S., Wang, P., Guo, J., Fang, Z., et al. (2022). More Positive or More Negative? Metagenomic Analysis Reveals Roles of Virome in Human Disease-Related Gut Microbiome. Front Cell Infect Microbiol *12*. 10.3389/FCIMB.2022.846063.

S53. Chen, Q., Ma, X., Li, C., Shen, Y., Zhu, W., Zhang, Y., Guo, X., Zhou, J., and Liu, C. (2021). Enteric Phageome Alterations in Patients With Type 2 Diabetes. Front Cell Infect Microbiol *10*. 10.3389/FCIMB.2020.575084.

S54. Ma, Y., You, X., Mai, G., Tokuyasu, T., and Liu, C. (2018). A human gut phage catalog correlates the gut phageome with type 2 diabetes. Microbiome *6*, 1–12. 10.1186/S40168-018-0410-Y/FIGURES/6.

S55. Fan, G., Cao, F., Kuang, T., Yi, H., Zhao, C., Wang, L., Peng, J., Zhuang, Z., Xu, T., Luo, Y., et al. (2023). Alterations in the gut virome are associated with type 2 diabetes and diabetic nephropathy. Gut Microbes *15*, 2226925. 10.1080/19490976.2023.2226925/SUPPL_FILE/KGMI_A_2226925_SM4471.ZIP.

S56. Han, M., Yang, P., Zhong, C., and Ning, K. (2018). The Human Gut Virome in Hypertension. Front Microbiol *9*. 10.3389/FMICB.2018.03150/FULL.

S57. Lang, S., Demir, M., Martin, A., Jiang, L., Zhang, X., Duan, Y., Gao, B., Wisplinghoff, H., Kasper, P., Roderburg, C., et al. (2020). Intestinal Virome Signature Associated With Severity of Nonalcoholic Fatty Liver Disease. Gastroenterology *159*, 1839–1852. 10.1053/J.GASTRO.2020.07.005.

S58. Nishijima, S., Nagata, N., Kiguchi, Y., Kojima, Y., Miyoshi-Akiyama, T., Kimura, M., Ohsugi, M., Ueki, K., Oka, S., Mizokami, M., et al. (2022). Extensive gut virome variation and its associations with host and environmental factors in a population-level cohort. Nature Communications 2022 13:1 *13*, 1–14. 10.1038/s41467-022-32832-w.

S59. Manrique, P., Zhu, Y., van der Oost, J., Herrema, H., Nieuwdorp, M., de Vos, W.M., and Young, M. (2021). Gut bacteriophage dynamics during fecal microbial transplantation in subjects with metabolic syndrome. Gut Microbes *13*, 1–15. 10.1080/19490976.2021.1897217/SUPPL_FILE/KGMI_A_1897217_SM5623.DOCX.

S60. Sandoval-Vargas, D., Concha-Rubio, N.D., Navarrete, P., Castro, M., and Medina, D.A. (2021). Short communication: Obesity intervention resulting in significant changes in the human gut viral composition. Applied Sciences (Switzerland) *11*, 10039. 10.3390/APP112110039/S1.
