# Supplementary material for: Bacteriophages, gut bacteria, and microbial pathways interplay in cardiometabolic health
Source: Cell Rep. 2024 Jan 31;43(2):113728. doi: 10.1016/j.celrep.2024.113728 (PMC11554570; doi:10.1016/j.celrep.2024.113728)
Supplement: Document S2. Article plus supplemental information [file mmc2.pdf]

Cell Reports

CellPress
OPEN ACCESS

Review

Bacteriophages, gut bacteria, and microbial pathways interplay in cardiometabolic health

Daniel Kirk,[1] Ricardo Costeira,[1] Alessia Visconti,[1,2] Mohammadali Khan Mirzaei,[3,4] Li Deng,[3,4] Ana M. Valdes,[5] and Cristina Menni[1,*]
[1]Department of Twin Research & Genetic Epidemiology, King's College London, St Thomas Hospital, Westminster Bridge Road, London SE1 7EH, UK
[2]Center for Biostatistics, Epidemiology, and Public Health, Department of Clinical and Biological Sciences, University of Turin, Turin, Italy
[3]Institute of Virology, Helmholtz Centre Munich - German Research Centre for Environmental Health, 85764 Neuherberg, Germany
[4]School of Life Sciences, Technical University of Munich, 85354 Freising, Germany
[5]Academic Rheumatology, Clinical Sciences Building, Nottingham City Hospital, University of Nottingham, Nottingham, UK
*Correspondence: cristina.menni@kcl.ac.uk
https://doi.org/10.1016/j.celrep.2024.113728

SUMMARY

Cardiometabolic diseases are leading causes of mortality in Western countries. Well-established risk factors include host genetics, lifestyle, diet, and the gut microbiome. Moreover, gut bacterial communities and their activities can be altered by bacteriophages (also known simply as phages), bacteria-infecting viruses, making these biological entities key regulators of human cardiometabolic health. The manipulation of bacterial populations by phages enables the possibility of using phages in the treatment of cardiometabolic diseases through phage therapy and fecal viral transplants. First, however, a deeper understanding of the role of the phageome in cardiometabolic diseases is required. In this review, we first introduce the phageome as a component of the gut microbiome and discuss fecal viral transplants and phage therapy in relation to cardiometabolic diseases. We then summarize the current state of phageome research in cardiometabolic diseases and propose how the phageome might indirectly influence cardiometabolic health through gut bacteria and their metabolites.

INTRODUCTION

In the last decades, there has been a dramatic worldwide increase in obesity and cardiometabolic diseases (CMDs) including type 2 diabetes mellitus (T2D), hypertension, cardiovascular disease (CVD), and nonalcoholic fatty liver disease (NAFLD).[1] CMDs are multifactorial disorders, and traditional risk factors include environmental exposures, diet, lifestyle, and genetic and epigenetic factors.[1] Recently, the gut microbiome has also emerged as a crucial player in CMDs, influencing various aspects of metabolic function and disease development.[2,3] Indeed, gut bacteria regulate multiple host functions, including digestion, immunity, and endocrine function.[4]

Despite a huge research investment in the gut microbiome over the last decade, there are still some unanswered questions, conflicting results, and a paucity of gut microbiome-based therapies.[5] Furthermore, gut microbiome research has been heavily biased toward the study of bacteria, when, in fact, viruses, archaea, and fungi are also present.[6] These components both modulate bacterial populations and interact with human health directly.[6] Specifically, bacteriophages (or simply, phages), bacteria-infecting viruses, are key drivers of bacterial community structure and function[7] and have thus been associated with not only gastrointestinal diseases but also systemic health, including CMDs.[8–12] By infecting gut bacteria, phages can (1) increase or decrease bacterial abundances[13] and (2) alter the function of their bacterial hosts even if the population numbers of the host or the phage remain unchanged.[14] This suggests that phages are indirectly associated with gut microbiome-associated diseases such as inflammatory bowel disease (IBD),[9] irritable bowel syndrome,[8] T2D[11] and the metabolic syndrome (MetS).[10]

Because of their capacity to modulate bacterial composition and function in the gut microbiome, phages have been considered to be therapeutic options in disease states in which gut bacteria are known to play a role.[7] Indeed, they have a narrow target host range, can remain active long after administration, and typically have minimal side effects or safety concerns for human hosts.[15] However, a deeper understanding of their role in the gut microbiome and human health is required to enable therapeutic breakthroughs. Two therapies in which phages play a fundamental role are fecal virome transplantation (FVT), which has already been applied in T2D and obesity[16–18] and phage therapy, which has been applied to various conditions, including gastrointestinal diseases, urinary tract infections, and antibiotic-resistant infections.[19,20] However, the extent to which

 © 2024 The Author(s).
This is an open access article under the CC BY license (http://creativecommons.org/licenses/by/4.0/).

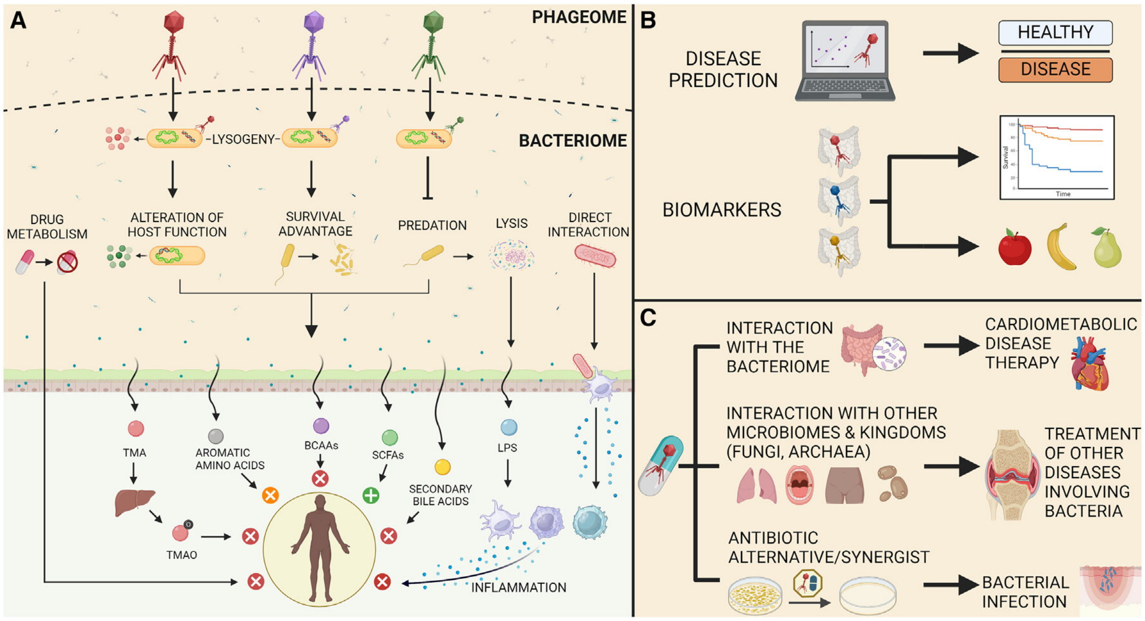

Figure 1. The gut phageome: Interactions, research applications, and therapeutic potentials
(A) Interactions between bacteria, phages, microbially derived metabolites, and human health.
(B) Applications of phages in research.
(C) Public health applications and therapeutic potential of phages.

the phages are involved in CMDs and whether the aforementioned therapies represent viable options for their treatment are currently unknown.

In this review, we introduce the phageome as a component of the gut microbiome. We then provide an overview of its role in human health, with a focus on cardiometabolic health and the potential for FVT and phage therapy, with examples from clinical and preclinical models. We then discuss the current state of phage research in CMDs and the links with the bacterial component of the gut microbiome. A deeper understanding of the role of phages in CMDs through phageome research can lead to novel mechanistic understandings and therapeutic breakthroughs.

THE PHAGEOME AS A COMPONENT OF THE GUT MICROBIOME

The gut microbiome and its components

The gut microbiome refers to the collection of bacteria, viruses (including phages), fungi, archaea, and their genes that exist in the digestive tract. These biological entities coexist in harmony with the host, and perturbations of the gut microbiome can lead to negative health outcomes (Figure 1).[7]

Colonization of the gut microbiome starts at birth.[21] Then, different factors shape its composition, including breastfeeding, use of antibiotics and medications, diet, and environmental exposures. Importantly, however, although the early-life influence is particularly strong, the gut microbiome reacts to changes at any point in the lifespan.[21] This plasticity combined with the relationship with health outcomes has made the gut microbiome the center of much research over the last decade.[22]

Most studies so far have focused on the bacterial component of the gut microbiome, partly because 16S rRNA gene amplicon sequencing has enabled the precise and sensitive detection of bacteria due to its ubiquity within the kingdom.[23] However, technological advances such as whole metagenome shotgun sequencing are also able to incorporate nonbacterial organisms in their analyses, including viral species.[24]

The phageome

The community of viruses that reside in the gut is mainly composed of phages (the phageome). Phage density increases along the gastrointestinal tract and reaches a maximum of 10^8–8^{10} phage virions per gram of feces in the large intestine.[25] Despite their abundance and potential roles in shaping gut microbiome composition and function,[25] phages remain largely uncharacterized, with up to 90% of all viral sequences in databases unknown (the "viral dark matter").

Phages can be broadly categorized based on their lifestyles, namely lysis and lysogeny[26] (Figure 2). Virulent phages have a lytic life cycle in which infection of their bacterial hosts is followed by DNA replication and lysis of the bacterium, causing the release of newly synthesized virions.[26] This affects bacterial production in the gut and can shift gut bacterial composition.[27] Phages are crucial in maintaining high prokaryotic richness in one environment by preferentially targeting the most abundant species of bacteria (the "kill-the-winner" hypothesis[28]). Conversely, in the gut, where microbial abundance and growth rates are high, phages may prefer to adopt temperate behavior and enter the lysogenic lifestyle ("piggyback-the-winner").

Lysogeny involves integrating the genetic material of the phage into the bacterial genome,[26] thus becoming a prophage

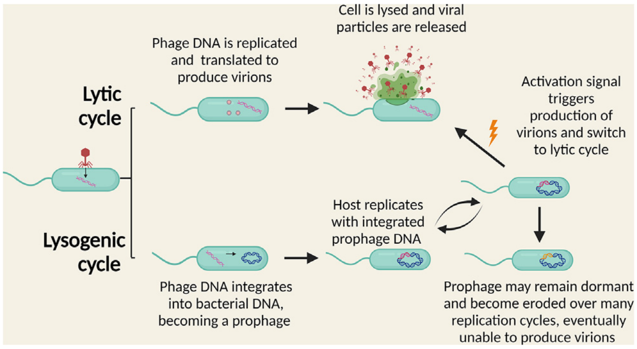

Figure 2. Life cycles of phages

that is automatically replicated and maintained when the bacterial cell replicates.[26] The prophage may stay inactive across many rounds of replication, usually requiring an activation signal within the host cell (e.g., stress) to become transcribed and translated, resulting in the release of newly formed virions.[25] Although this can slow bacterial production since resources are directed away from the bacterium and toward virion synthesis,[26] bacterial fitness is often increased in a process resembling symbiosis.[27] Alternatively, prophage DNA may be altered due to accumulated mutations over many bacterial reproduction cycles, eventually losing its ability to be transcribed and produce virions.[25]

During their life cycles, phages have the capacity to transfer genes from one bacterial species to another (horizontal gene transfer).[27–29] This is done via processes of transduction that can be either generalized (i.e., a random piece of the host DNA is incorporated during cell lysis) or specialized (i.e., a prophage imprecisely excises itself from a host genome and incorporates some of the flanking host DNAs into their own[29]). Transduction has the potential to modulate the fitness of the bacterial hosts of the phages.[27–29] For instance, phage-encoded auxiliary metabolic genes (AMGs) originated from bacteria and then incorporated into the phage genome, are pervasively found in phages.[30] AMGs can alter metabolic processes in the gut by reprogramming bacterial host metabolism and encoding bacterial exotoxins.[30] As such, AMGs and phages encoding them have been associated with immunomodulation, lipopolysaccharides (LPSs), and intestinal permeability, all of which are associated with CMDs.[30]

Phage bioinformatics

Historically, phageome characterization relied on cultivation-based methods, including isolating viral-like proteins (VLPs) from ecological environments and enriching host bacteria. These methods have led to the isolation of phages from certain model hosts,[31] and a recent large-scale phage cultivation study has successfully isolated 209 phages for 42 commensal human gut bacterial species.[32] However, although cultivation studies facilitate bacterial phage-host assignment and do not rely on reference databases, they remain limited in scale. They are also restricted to gut bacteria that can be cultured *in vitro*. Hence, bioinformatic methods are the most common and effective approaches for characterizing the gut phageome. Recent advances in metagenomic sequencing and computational tools for the analysis of data thereof have enabled a more in-depth analysis of the complexity and richness of phages in the gut microbiome.[14,33,34]

Metagenomic sequencing involves first the extraction of nucleic acid material from a sample (e.g., feces), which may be total DNA, only the viral fraction, or both.[35] A library is then prepared from which the extracted genetic material is sequenced, followed by quality control steps on the raw reads.[24] Reads remaining after quality control are then annotated by mapping reads to an existing bacteriophage database or by *de novo* assembly. Mapping reads to a reference database allows the instant identification of the species present in a sample; however, these approaches are limited to the taxonomic information available in the reference databases used.[24] However, *de novo* assembly, being independent of reference databases, enables a much more complete picture of the phageome but is sensitive to the software used for assembly.[28] Pipelines including MetaPhlAn4[34] and ViroProfiler[36] estimate phageome composition from shotgun metagenome data. The latest release of MetaPhlAn4[34] includes over 162,000 viral sequences, whereas ViroProfiler[36] is a containerized metagenomic data analysis tool with capabilities such as viral discovery, taxonomy assignment, functional annotation, and host and replication cycle predictions.

These technological advances have led to a better characterization of the phage component of the microbiome.[27] Among the most common and well-studied phages in the gut, there are (1) the order Caudovirales, with double-stranded DNA (dsDNA) genomes and a tail structure,[28] and (2) the *crAssphages*, so named due to the cross-assembly of phage sequencing data.[37] *crAssphages* have unique genetic sequences that make up a significant portion (up to 90%[37]) of VLP-derived metagenomes in some populations, including Western, Korean, and Malawian.[37,38] In addition, *crAssphages* bear no resemblance to any known phages and have been detected in more than 50% of the gut phageomes of the Western population,[39] are associated with industrialization, and both positive and negative associations have been found with obesity across its various subfamilies.[40,41]

Predicting bacterial hosts

Because phages modulate the gut bacteria composition, it is important to understand which bacteria they infect. Various approaches are involved in predicting bacterial hosts of phages.[42] These include comparing genetic homology between phages and bacteria and investigating phage-host abundance profiles.

Homology between phage and bacterial genetic sequences can reveal previous infection events between a phage and a bacterial species and can arise due to horizontal transfer, prophage integration, insertion sites (e.g., tRNAs), or CRISPR spacer sequences.[42,43] For example, CRISPR spacers have been used to identify the hosts of thousands of newly discovered phages from whole-community metagenomes in the NCBI Assembly database, with hosts including the *Bacteroides* genera, implicated in CMDs,[44] and whose fitness was influenced by the phages infecting them.[45] In contrast, phage-host abundance methods are based on the idea that the interaction dynamics

of phages and bacteria can be used to assign bacterial hosts to phages.[46] With data obtained from repeated sampling, these correlation-based approaches aim to assign hosts by analyzing common trends between phages and bacteria in an environment.[43,46] In doing so, assumptions are made about the relationships between phages and bacteria, which, given the complex dynamics of the gut microbiome, may lead to bias.[43,46,47]

MANIPULATING THE GUT MICROBIOME: PHAGE THERAPY AND FVT

FVT and phage therapy are two different approaches that involve the use of viruses and phages to modify gut microbiome composition and potentially gain therapeutic benefits. FVT involves the transfer of viral components from the stool of a healthy donor to the gut of a recipient to restore the microbiome.[48] Phage therapy, conversely, uses a targeted approach to isolate and transplant phages that are effective against the specific bacterial strain causing the infection.[15]

Fecal virome transplant

Unlike fecal matter transplantation (FMT), which transfers a wide range of microorganisms, FVT specifically targets the virome by transferring only viral components (including phages) from fecal matter. The fecal matter of the donor is first treated to remove intact bacterial cells via a size exclusion filter[49] and then transplanted into the recipient with the aim of manipulating bacterial populations.[7] Metabolites, macronutrients, and bacterial cell wall components will, however, remain in the fecal matrix.[50] FVT has the advantage of reducing risks associated with FMTs such as bacterial infection.[7]

In preclinical studies, a reduction in weight and an improvement in oral glucose tolerance,[16,18] liver pathology, adipose inflammation, and glucose clearance[51] were observed in (high-fat diet-induced) obese mice receiving FVT from lean donor mice. Although FVTs have not yet been applied to CMD in humans, FMTs, which lead not only to the transplantation of bacteria but also of viruses and phages, have. Changes in the phageome following FMTs have been reported for various conditions, including *Clostridium difficile* infection, IBD, and autism spectrum disorder, among others.[52] In a recent study, Manrique and colleagues transplanted fecal matter from five healthy donors to six individuals with MetS and observed a significant change in the gut phageomes of the recipients.[17] Phageome richness and similarity between the phageome in the recipient post-transplantation and the donor phageome were correlated to FMT success.[17] Although the sample size was small and it was not possible to assess whether phage community changes were a driving force in reshaping the gut microbiome post-transplant or simply a secondary reaction to bacterial changes, the study suggests that changes in the phageome correlate with clinical outcomes, including CMD.[17]

FVT challenges

Whereas FVTs pose potential advantages to FMTs due to the elimination of the bacterial components and thus the transmission of unwanted pathogens, the current lack of studies in this area prevents a deeper understanding of their effects, especially in the long term.[49] In addition, unlike traditional medicines or antibiotics, phage-based treatments such as FVT involve the use of biological agents that could remain active in the gut microbiome of the recipient indefinitely.[15,53] Although this could lead to long-term treatment success, co-occurring side effects due to the treatment may also persist.

Another significant challenge to the FVT is the transfer of unwanted viruses that are known to reside in the gut (e.g., herpesvirus) from the donor to the recipient with unknown consequences upon transmission.[54] A thorough screening of the donor virome would therefore be needed before transplantation to characterize the viral species and the encoded genes.[54] Alternatively, the filtrate would need to be modified before transplantation by using a solvent treatment to inactivate enveloped (eukaryotic) viruses,[51] leaving most of the phage community intact. Still, even with eukaryotic viruses eliminated, much of the phageome remains uncharacterized,[55] which motivates the need for a better understanding of the gut phageome in human health.

Phage therapy

A key property of phages is their ability to target specific bacteria while leaving off-target species largely unaffected, which has led to their use as a treatment option known as phage therapy.[15] Phage therapy involves using phages to target specific bacteria involved in disease progression.[15] Recent research findings suggest the use of phages as an alternative to antibiotics due to their narrower target range and fewer off-target effects on other bacterial species, minimal side effects on human hosts, and their ability to coevolve with their bacterial hosts.[15] These theoretical advantages of using phages have motivated efforts such as the Centre for Phage Research in Leicester, UK, which provides a biobank repository and plans to host a national library of phages to facilitate efforts in phage therapy.[56]

Although phage therapy has not yet been applied in CMDs, research on its application in other diseases can be informative. For instance, a recent systematic review of 27 studies and 165 patients supported the efficacy and safety of phage therapy in the treatment of infectious diseases caused by various multi-drug-resistant strains of bacterial species.[57] This included using phage therapy to target *Escherichia coli, Klebsiella pneumoniae*, and *Streptococcus*,[57] all of which are known to be associated with several CMDs.[58–61]

Moreover, promising results were reported in the double-blinded crossover PHAGE study investigating the effect of a combination of phages targeting *E. coli* in participants with gastrointestinal complaints.[62] Indeed, a significant reduction in *E. coli* populations was reported, whereas the non-target populations remained largely unchanged.[62] In addition, preclinical studies consistently supported the safety of phage therapy and reported minimal effects on commensal bacteria.[15]

Phage therapy challenges

Because CMDs are usually characterized by a general dysbiosis and not by the dominance of a particular species,[63] phage therapy in the treatment of CMDs may involve the development of a phage cocktail. Moreover, there are still challenges around the mode of delivery, the dosage, the stability of phage preparations (ensuring that the administered phages can reach their target location and infect their target host), and the ethical implications of using phage therapy as a treatment.[64]

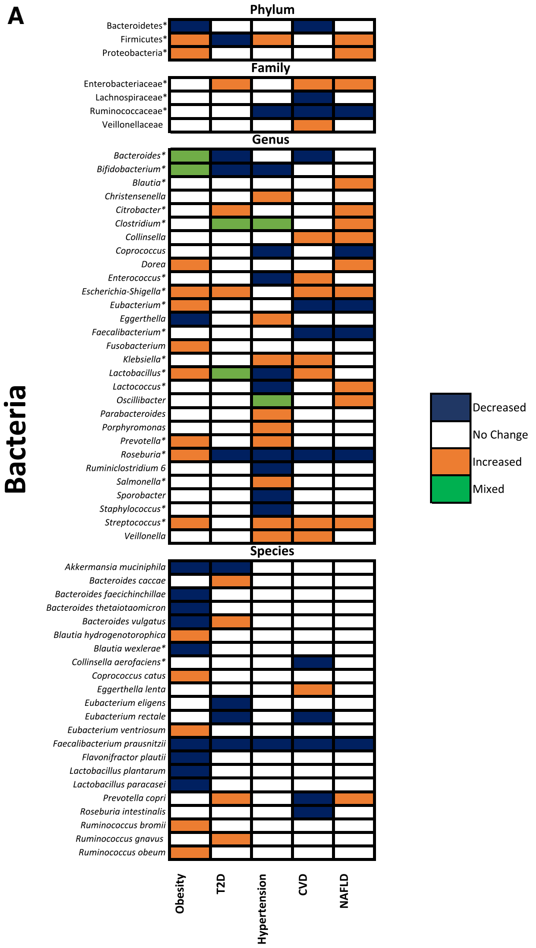

B

Microbially-derived Metabolite	Effect on CMD	Associated CMDs	Associated bacteria
Short-chain fatty acids	↑ Beneficial	Obesity, T2D, NAFLD, hypertension, CVD	**Butyrate:** *Roseburia, Anaerostipe, Faecalibacterium prausnitzii, Eubacterium hallii, Eubacterium rectale, Clostridium leptum* **Acetate:** *Bifidobacterium*†, *Prevotella, Ruminococcus*†, *Bacteroides*†, *Clostridium*†, *Streptococcus*†, *Akkermansia muciniphila, Blautia hydrogenotrophica* **Propionate:** *Negativicutes* (Firmicutes), *Bacteroides*†, *Ruminococcus*†, *Blautia, Akkermansia mucinihila*†, *Coprococcus catus, Roseburia inulinivorans*
Trimethylamine	↓ Harmful	CVD	**TMA:** *Anaerococcus hydrogenalis, Clostridium asparagiforme, Clostridium hathewayi, Clostridium sporogenes, Edwardsiella tarda, Escherichia fergusonii, Proteus penneri, Providencia rettgeri*
Secondary bile acids	↓ Harmful	T2D, NAFLD CVD	**Deconjugation:** *Bacteroides*†, *Bifidobacterium*†, *Lactobacillus*†, *Clostridium*†, *Enterococcus*†, *Listeria*†, *Stenotrophomonas, Brucella* **Dehydroxylation:** *Clostridium scindens, Clostridium hylemonae, Peptacetobacter hiranonis* **Oxidation & epimerization:** *Ruminococcus gnavus, Clostridium absonum, Stenotrophomonas maltophilia, Collinsella aerofaciens*†, *Blautia producta, Eggerthella lenta, Clostridium baratii, Enterorhabdus mucosicola*
Branched chain amino acids	↓ Harmful	Obesity, T2D, CVD	**BCAA:** *Clostridium*†, *Peptostreptococci, Streptococcus*†, *Prevotella, Bacteroides*†, *Klebsiella*†, *Escherichia coli, Selenomonas ruminantium, Megasphaera elsdenii, Staphylococcus aureus*
Aromatic amino acid metabolites	(↓) Mostly Harmful	T2D, NAFLD CVD	**p-cresol:** *Coriobacteriaceae, Clostridium clusters XI and XIVa*† **Indole:** *Shigella*†, *Escherichia coli, Escherichia faecalis, Klebsiella planticola, Proteus vulgaris*, other pathogenic bacteria **Phenylacetylglutamine:** *Bacteroides thetaiotaomicron, Proteus mirabilis, Clostridium sporogenes*
LPS	(↓) Mostly Harmful	Obesity, T2D, NAFLD, CVD	**LPS:** Gram-negative bacteria†

Figure 3. Gut bacteria, microbial metabolites, and CMD associations
(A) Bacteria associated with CMDs by taxonomic rank. Orange indicates a positive association, blue indicates a negative association, white indicates a lack of association, and green indicates discordant results. The asterisk indicates that phages have been identified for the bacteria as reviewed here.
(B) Major microbially derived metabolites, their associated bacteria, and their effect on cardiometabolic health. The cross symbol signifies bacteria for which phages have also been identified in the studies described in the text. Due to the variety of aromatic amino acid metabolites and their associated bacteria, only a few key examples are provided. Parentheses around an arrow convey that most of the compounds in a given class have the effect represented by the arrow, although some may also have the opposite effect, as is the case with certain aromatic amino acid metabolites and some LPS molecules.

THE ROLE OF PHAGES IN CMDs

To maximize the potential of phage-based therapies, a deeper understanding of how the phageome is altered in CMDs and how this relates to the bacterial component of the gut microbiome is required. Research in this area is limited but continuing to grow and is discussed in detail below.

Gut bacteria

Gut bacteria composition and diversity, as well as gut microbiome function, have been consistently implicated in CMDs, including obesity, T2D, hypertension, CVD, and NAFLD, among others, as summarized in Figure 3A and Table S1. For instance, a reduction in gut microbiome diversity and *Ruminococcaceae*, *Roseburia*, *Faecalibacterium Prausnitzii*, and *Akkermansia*, and an increase in *Enterobacteriaceae*, *Escherichia-Shigella*, *Klebsiella*, *Lactobacillus*, and *Streptococcus* have been reported in individuals with CMDs[4] (Figure 3A). There are several mechanisms whereby the gut microbiome influences human health, including affecting gut permeability and thus regulating excretion and absorption; affecting inflammation through the activation of immune cells and the production of proinflammatory and anti-inflammatory signaling molecules; affecting neurotransmitter production and hormonal regulation; and, especially, by producing bacterial metabolites, including short-chain fatty acids, secondary bile acids, and branch chain amino acids. Bacterial metabolites are released into the bloodstream and are responsible for several metabolic conditions, including insulin resistance, diabetes, and obesity, thus mediating microbial effects on human health[65] (see Figure 3B; Table S2).

Bacterial metabolites are indeed the key agents involved in the role of the gut microbiome in CMD. For instance,

butyrate-producing bacteria are associated with a lower risk of T2D,[66] acetate has been shown to mediate the effect of gut bacteria composition on visceral fat, and the gut bacteria-produced secondary bile acid isoursodeoxycholate is associated with liver function, postprandial lipemia, and inflammation.[67]

Gut phageome

In contrast to the bacterial component of the gut, studies investigating the role of the gut phageome in CMDs are still in their infancy. Results are not always consistent across different disease states, and studies tend to be underpowered and have small sample sizes (see Figure 4; Table S3). Below, a summary of the existing studies by CMD is provided.

Obesity

A different phageome profile was observed in individuals with obesity compared to healthy controls or individuals with other CMDs.[11,60,68] Differences in phages were also observed in 49 individuals before and after obesity intervention treatment (e.g., surgery, diet, exercise)[69] and in obese children compared to normal-weight controls in *crAssphage* subfamilies[41] (a taxonomic rank between the family and genus levels[70]). Moreover, a large study including 4,198 Japanese individuals reported a positive correlation between dsDNA phage diversity and BMI,[71] whereas results were somewhat inconsistent in other smaller studies (n < 229).[11,68,69]

T2D

Individuals with T2D have been found to present differences in gut phageome composition compared to healthy controls.[11,72–74] This includes a higher abundance of common phage operational taxonomic units (i.e., those present in over two-thirds of the sample),[73] *Shigella* and *Xylella* phages[74]; *Enterobacteria phage cdtl*, *Enterobacteria phage ES18*, *Klebsiella phage KP34*, *Salmonella phage ST64T*[72]; *Cellulophaga phage and Bacteroides phage*[72]; lower levels of *Flavobacterium*, *Cellulophage*, *Staphylococcus*, *Synechoccus*, *Curvibacter*, *Clostridoides*, *Tenacibaculum*, *Paenibacillus*, *Lactobacillus*, *Listeria*, and *Citrobacter* phages[74]; *Brochothrix phage A9*, *Brochothrix phage NF5*, *Enterococcus phage phiFL2A*, and *Salmonella phage* PVP-SE1[72]; and *Thermoanaerobicbacterium phage*, *Verrucomicrobia phage*, and *Proteus phage*[11]; and alterations at the family level in individuals with T2D as compared to controls.[73,74] In addition, the use of diabetes medication was found to correlate to dsDNA phageome composition.[71] Chen and coworkers observed that certain phages, including *Bacillus*, *Enterococcus*, *Streptococcus*, and *Klebsiella*, correlated with fasting blood glucose and insulin, postprandial insulin, highly sensitive C-reactive protein, and free thyroxine.[72] Furthermore, the gut phageome of individuals with T2D appeared more perturbed than the gut phageome of individuals with obesity when compared to healthy controls.[11] All of the differences in phages observed in those with obesity were also observed in those with T2D but not vice versa. Phageome richness and diversity were reported to be lower in individuals with T2D,[74] although results are inconsistent across studies.[11,71–73]

MetS

Studies report compositional alterations in individuals with MetS.[10,41,68,75] For instance, a recent study in 196 Dutch individuals reported that individuals with MetS have a higher abundance of *Bacteroidaceae*- and *Streptococcaceae*-infecting phages and a lower abundance of *Bifidobacteriaceae*-infecting phages compared to healthy controls.[10] In addition, a new phage family, called *Candidatus Heliusviridae*, was present in more than 96% of participants and had subfamilies that were related to MetS.[10] The Crassvirales order was also significantly less prevalent in the MetS phageomes compared to controls.[10] Individuals with MetS also have a different relative abundance of *crAssphage* compared to healthy controls, although the direction of the association is not clear.[41,68,75]

In contrast to the results found in T2D,[73] highly prevalent phages appear to be reduced in individuals with MetS.[10,68] De Jonge and coworkers identified two viral clusters present in >30% of controls, whereas these were not present in MetS, and there were no viral clusters present in >30% of the individuals in MetS.[10] Similarly, Bikel et al. found that the average prevalence of highly abundant (present in >80%) phage contigs in the normal-weight group decreased from 91.54% to 76.35% in obesity and to 68.27% in MetS.[68]

A very small study reported an increase in richness and diversity in schoolchildren with obesity and MetS compared to healthy controls.[68] In contrast, in a larger study in adults, lower richness and diversity were observed in those with MetS.[10] Phage richness was also negatively correlated with obesity, blood glucose, blood pressure, and triglycerides.[10]

Hypertension

Han and colleagues found that dominant phages across levels of hypertension were different.[76] For instance, the gut phageome of individuals with hypertension was reported to be dominated by *Klebsiella phage KP32*, *Cyanophage S-TIM5*, and *Salmonella phage FSL SP-004*; that of individuals with prehypertension by *Cronobacter phage CR3*, *Cronobacter phage ENT39118*, and *Cronobacter phage phiES15*; and finally, *Salmonella phage vB-SemP-Emek*, *Pseudomonas phage PaMx11*, and *Gordonia phage GTE8* dominated the gut phageome of controls.[76] No difference in diversity was observed across the groups. In addition, the dsDNA phageomes of 4,198 Japanese individuals were associated with hypertension.[71] However, due to the limited number of studies, more research is required to understand the role of the phageome in hypertension, including research investigating the relation of the phageome to blood pressure as a continuous outcome.

NAFLD

Only one study investigated the phageome in NAFLD,[12] in which a reduction in *Lactococcus* and an increase in *Streptococcus* phages in VLP fractions in NAFLD patients with higher NAFLD activity scores (NAS; a measure of NAFLD severity based on histological lesions of the liver[77]) was reported.[12] Moreover, VLPs from patients with NAS of 5–8 had lower phageome diversity and a lower proportion of phage to nonphage genetic material compared with those with NAS 0–4 or controls.[12] More studies investigating phageome alterations in NAFLD are required to validate these results.

CVD and hypertriglyceridemia

Jie and colleagues reported that individuals with atherosclerotic CVD have a different phageome profile compared to healthy controls with an enrichment in *Enterobacteriaceae*- and *Streptococcus*-infecting phages.[60] They observed that individuals with

CellPress
OPEN ACCESS

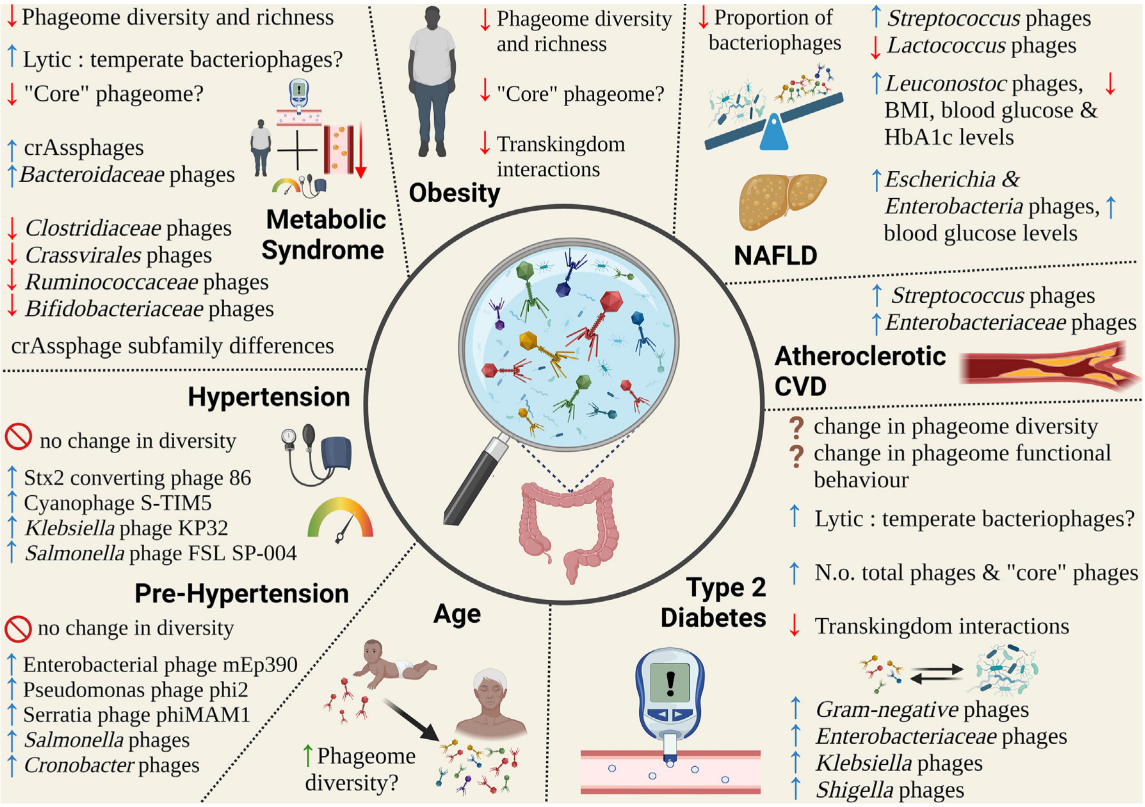

Figure 4. Association between the human phageome and CMDs

atherosclerotic CVD had a phageome profile similar to that of individuals with cirrhosis, whereas little overlap was observed with those suffering from obesity, T2D, and rheumatoid arthritis.[60] The dsDNA phageome was also found to correlate with CMD medications, including platelet aggregation inhibitors and statins.[71]

POTENTIAL MECHANISMS OF LINKING THE PHAGEOME WITH CMD

Recent studies investigated the gut bacteria–phage relationship and reported alterations in phages infecting bacteria related to CMDs, including *Bacteroides*,[10,11,73,74] *Bifidobacterium*,[10] *Blautia*,[10] *Clostridium*,[10,73] *Escherichia-Shigella*,[11,69,72–74] *Lactobacillus*,[11,73,74] *Klebsiella*,[76] *Roseburia*,[10] and *Streptococcus*[11,12,60,72] (see Figure 3A).

In a study of 90 individuals with T2D and 42 healthy controls, Fan and colleagues identified several significant associations between phages and both short-chain fatty acid (SCFA)-producing bacteria (e.g., *F. prausnitzii, Roseburia faecis, R. inulinivorans*) and other bacteria with known relationships to CMDs (including *Akkermansia muciniphila*).[74] Some of the identified associations appeared to be disease-specific, because when stratifying by disease status, the phage–bacteria correlations were not always consistent. For instance, *Shigella* phage correlated with the genera *Bluatia*, *Bacteroides*, and *Clostridium* only in those with T2D, whereas *Pseudomonas phage* was found to correlate with *A. muciniphila* and *Ruminococcus bromii* only in controls.[74] The results suggest that transkingdom interactions are altered in T2D and shed light on how changes in gut bacteria co-occur with changes in gut phages.

Another study in T2D used network analysis to show that the bacterial genera with the most connections to phages were *Escherichia* and *Bacteroides*, two genera that are commonly associated with CMDs[73] (Figure 3A). Consistently, other studies report changes in phage–bacteria interactions, including a reduction in the number of correlations between phages and bacteria in obesity, T2D, and hypertension.[72,76] Besides influencing bacterial population numbers, phages can provide accessory genes via prophage integration and horizontal gene transfer, therefore influencing the metabolic activity of their hosts.[10,73]

A switch toward increased lytic phages or their activity has been suggested in T2D and MetS.[10,72,73] For example, Chen and colleagues sampled the extracellular phageome using VLPs in 17 diabetic patients and 29 nondiabetic controls and found a positive correlation between Gram-negative phages and their bacterial hosts, especially for *Enterobacteriaceae* and phages of members of this family, such as *Escherichia*, *Salmonella*, *Enterobacter*, *Shigella*, *Klebsiella*, and *Enterobacteria* phages.[72] It is thought that an elevation in Gram-negative bacteria and their phages causes a "lytic switch," leading to the release of bacterial cell components and inflammation, thus contributing to metabolic disease pathology.[72,78–80] LPS was

also elevated in the study of Chen and colleagues.[72] In further support of this, Ma et al. also report a positive relationship between *Enterobacteria* and *Escherichia* and their phages, which is elevated in T2D, and de Jonge and colleagues found lower intracellular phage-to-bacterial ratios and higher viral counts in the VLP fraction, both of which suggest a lower lysogenic phage behavior.[10,73] However, the results are somewhat in contrast to those of Fan and colleagues, who found an increased number of positive phage–bacterial correlations and a decreased number of negative ones in T2D, which would not be expected under increased lysis.[74] Taken together, it is currently unclear whether a lytic shift occurs in CMDs, although this could be an interesting hypothesis to explore in future research.

In conclusion, the available studies, albeit limited, show a role for the phageome in CMDs and support a role for phage-based therapies in the manipulation of the bacterial component of the gut microbiome. In phage therapy, this would involve engineering phages to be delivered to the gut, where they would modify the abundance of CMD-associated bacteria (e.g., by increasing the abundance of SCFA-producing bacteria) or modify their metabolic behavior. Due to the variability of microbial alterations across different CMDs (see Figure 3A), this will probably be done on a disease- or even case-specific basis, which increases the technical challenges and costs. In FVT, this would involve the identification of healthy donor phageomes to be used to restore CMD-associated bacterial dysbiosis. However, the potential irreversible transfer of undesirable viruses and the consequences thereof must be carefully considered if FVTs are to be used in the treatment of CMDs.

CONCLUSION AND FUTURE PERSPECTIVES

The emerging role of the phageome in CMDs gives rise to the possibility of using phages for diagnosis and treatment. The associations between phages and CMDs and other diseases suggest that phages represent a potential biomarker reservoir that may improve disease prediction and prognosis compared to traditional biomarkers or bacterial data alone. In terms of treatment, key properties of phages such as host specificity and minimal off-target effects[15] make them attractive options for altering the bacterial component of the gut microbiome, which is implicated in CMDs.[2] Although phage-based therapies and FVT are in their infancy, and randomized controlled trials (RCTs) supporting their use are limited,[17] their translational potential presents a promising avenue for addressing the pressing challenges in the realm of CMD treatment.

Technological advancements and modern bioinformatics techniques are leading to a better characterization of the gut phageome and its role in CMDs, and future work will certainly benefit from the rapidly evolving field of artificial intelligence, which will have applications such as improving the quality of metagenomic samples, annotating phages from these samples, and predicting bacterial hosts of the phages that are identified. To fully capitalize on these advantages, however, more work in larger samples is needed to improve the statistical power of tests of association between phages and bacteria or disease outcomes. In addition, RCTs and longitudinal evidence are needed to bridge the gap between basic research and clinical applications, and interactions across the diverse array of taxonomic kingdoms in the gut (archaea, bacteria, fungi) should be considered to provide a more complete picture of the gut microbiome landscape. Exclusively studying bacteria may fail to account for the role of phages in diseases with a nonbacterial etiology.

Moreover, there has been much research interest in using phage therapies for drug-resistant bacterial infection, particularly in the context of antibiotic resistance.[15,81] Because phages are highly specific, they leave off-target species, including beneficial bacteria, unaffected.[15] They may also work synergistically with traditional antibiotics, enhancing their efficacy.[81]

Finally, the advantages of phage therapies and FVT can be extended beyond bacterial infections to other diseases in which bacteria are involved, to other organ microbiomes (oral, skin, lung, and vaginal). Examples may include endometriosis[82,83] and the links between periodontitis and rheumatoid arthritis,[84,85] where oral and vaginal microbiome features are involved in symptoms.

SUPPLEMENTAL INFORMATION

Supplemental information can be found online at https://doi.org/10.1016/j.celrep.2024.113728.

ACKNOWLEDGMENTS

C.M. and D.K. are funded by the Chronic Disease Research Foundation. Support for this work was also provided by UKRI/MRC grants MR/W026813/1 and MR/Y010175/1 to C.M. and A.M.V.

AUTHOR CONTRIBUTIONS

Preparation, creation, and/or presentation of the published work, specifically writing the initial draft (including substantive translation) were done by D.K., A.M.V., and C.M. Preparation, creation, and/or presentation of the published work by those from the original research group, specifically critical review, commentary, or revision (including pre- or postpublication stages) were completed by R.C., M.K.M., A.M.V., and L.D. Preparation, creation, and/or presentation of the published work, specifically visualization/data presentation were done by D.K. and C.M.

DECLARATION OF INTERESTS

A.M.V. is a consultant for Zoe Global Ltd.

REFERENCES

1. World Health Statistics 2023: Monitoring Health for the SDGs (2023) (Sustainable Development Goals).
2. Valles-Colomer, M., Menni, C., Berry, S.E., Valdes, A.M., Spector, T.D., and Segata, N. (2023). Cardiometabolic health, diet and the gut microbiome: a meta-omics perspective. Nat. Med. *29*, 551–561. https://doi.org/10.1038/s41591-023-02260-4.
3. Dabke, K., Hendrick, G., and Devkota, S. (2019). The gut microbiome and metabolic syndrome. J. Clin. Invest. *129*, 4050–4057. https://doi.org/10.1172/JCI129194.
4. Fan, Y., and Pedersen, O. (2020). Gut microbiota in human metabolic health and disease. Nat. Rev. Microbiol. *19*, 55–71. https://doi.org/10.1038/s41579-020-0433-9.
5. Ilan, Y. (2019). Why targeting the microbiome is not so successful: can randomness overcome the adaptation that occurs following gut manipulation? Clin. Exp. Gastroenterol. *12*, 209–217. https://doi.org/10.2147/CEG.S203823.

CellPress
OPEN ACCESS

6. Vemuri, R., Shankar, E.M., Chieppa, M., Eri, R., and Kavanagh, K. (2020). Beyond Just Bacteria: Functional Biomes in the Gut Ecosystem Including Virome, Mycobiome, Archaeome and Helminths. Microorganisms *8*, 483. https://doi.org/10.3390/MICROORGANISMS8040483.

7. Cao, Z., Sugimura, N., Burgermeister, E., Ebert, M.P., Zuo, T., and Lan, P. (2022). The gut virome: A new microbiome component in health and disease. EBioMedicine *81*, 104113. https://doi.org/10.1016/J.EBIOM.2022.104113.

8. Liu, A., Gao, W., Zhu, Y., Hou, X., and Chu, H. (2022). Gut Non-Bacterial Microbiota: Emerging Link to Irritable Bowel Syndrome. Toxins *14*, 596. https://doi.org/10.3390/TOXINS14090596.

9. Norman, J.M., Handley, S.A., Baldridge, M.T., Droit, L., Liu, C.Y., Keller, B.C., Kambal, A., Monaco, C.L., Zhao, G., Fleshner, P., et al. (2015). Disease-specific alterations in the enteric virome in inflammatory bowel disease. Cell *160*, 447–460. https://doi.org/10.1016/J.CE. S. ALL.2015.01.002.

10. de Jonge, P.A., Wortelboer, K., Scheithauer, T.P.M., van den Born, B.J.H., Zwinderman, A.H., Nobrega, F.L., Dutilh, B.E., Nieuwdorp, M., and Herrema, H. (2022). Gut virome profiling identifies a widespread bacteriophage family associated with metabolic syndrome. Nat. Commun. *13*, 3594. https://doi.org/10.1038/S41467-022-31390-5.

11. Yang, K., Niu, J., Zuo, T., Sun, Y., Xu, Z., Tang, W., Liu, Q., Zhang, J., Ng, E.K.W., Wong, S.K.H., et al. (2021). Alterations in the Gut Virome in Obesity and Type 2 Diabetes Mellitus. Gastroenterology *161*, 1257–1269.e13. https://doi.org/10.1053/J.GASTRO.2021.06.056.

12. Lang, S., Demir, M., Martin, A., Jiang, L., Zhang, X., Duan, Y., Gao, B., Wisplinghoff, H., Kasper, P., Roderburg, C., et al. (2020). Intestinal Virome Signature Associated With Severity of Nonalcoholic Fatty Liver Disease. Gastroenterology *159*, 1839–1852. https://doi.org/10.1053/J.GASTRO.2020.07.005.

13. Mirzaei, M.K., and Maurice, C.F. (2017). Ménage à trois in the human gut: interactions between host, bacteria and phages. Nat. Rev. Microbiol. *15*, 397–408. https://doi.org/10.1038/nrmicro.2017.30.

14. Khan Mirzaei, M., and Deng, L. (2022). New technologies for developing phage-based tools to manipulate the human microbiome. Trends Microbiol. *30*, 131–142. https://doi.org/10.1016/J.TIM.2021.04.007.

15. Gordillo Altamirano, F.L., and Barr, J.J. (2019). Phage Therapy in the Post-antibiotic Era. Clin. Microbiol. Rev. *32*, e00066-18. https://doi.org/10.1128/CMR.00066-18.

16. Rasmussen, T.S., Mentzel, C.M.J., Kot, W., Castro-Mejía, J.L., Zuffa, S., Swann, J.R., Hansen, L.H., Vogensen, F.K., Hansen, A.K., and Nielsen, D.S. (2020). Faecal virome transplantation decreases symptoms of type 2 diabetes and obesity in a murine model. Gut *69*, 2122–2130. https://doi.org/10.1136/GUTJNL-2019-320005.

17. Manrique, P., Zhu, Y., van der Oost, J., Herrema, H., Nieuwdorp, M., de Vos, W.M., and Young, M. (2021). Gut bacteriophage dynamics during fecal microbial transplantation in subjects with metabolic syndrome. Gut Microb. *13*, 1–15.

18. Borin, J.M., Liu, R., Wang, Y., Wu, T.-C., Chopyk, J., Huang, L., Kuo, P., Ghose, C., Meyer, J.R., Tu, X.M., et al. (2023). Fecal virome transplantation is sufficient to alter fecal microbiota and drive lean and obese body phenotypes in mice. Preprint at bioRxiv. https://doi.org/10.1101/2023.02.03.527064.

19. Duan, Y., Young, R., and Schnabl, B. (2021). Bacteriophages and their potential for treatment of gastrointestinal diseases. Nat. Rev. Gastroenterol. Hepatol. *19*, 135–144. https://doi.org/10.1038/s41575-021-00536-z.

20. Divya Ganeshan, S., and Hosseinidoust, Z. (2019). Phage Therapy with a Focus on the Human Microbiota. Antibiotics *8*, 131. https://doi.org/10.3390/ANTIBIOTICS8030131.

21. Senn, V., Bassler, D., Choudhury, R., Scholkmann, F., Righini-Grunder, F., Vuille-dit-Bile, R.N., and Restin, T. (2020). Microbial Colonization From the Fetus to Early Childhood—A Comprehensive Review. Front. Cell. Infect. Microbiol. *10*, 573735. https://doi.org/10.3389/FCIMB.2020.573735/BIBTEX.

22. Huang, Z., Liu, K., Ma, W., Li, D., Mo, T., and Liu, Q. (2022). The gut microbiome in human health and disease—Where are we and where are we going? A bibliometric analysis. Front. Microbiol. *13*, 1018594. https://doi.org/10.3389/FMICB.2022.1018594/BIBTEX.

23. Ames, N.J., Ranucci, A., Moriyama, B., and Wallen, G.R. (2017). The Human Microbiome and Understanding the 16S rRNA Gene in Translational Nursing Science. Nurs. Res. *66*, 184–197. https://doi.org/10.1097/NNR.0000000000000212.

24. Garmaeva, S., Sinha, T., Kurilshikov, A., Fu, J., Wijmenga, C., and Zhernakova, A. (2019). Studying the gut virome in the metagenomic era: challenges and perspectives. BMC Biol. *17*, 84. https://doi.org/10.1186/S12915-019-0704-Y.

25. Zuppi, M., Hendrickson, H.L., O'Sullivan, J.M., and Vatanen, T. (2021). Phages in the Gut Ecosystem. Front. Cell. Infect. Microbiol. *11*, 822562. https://doi.org/10.3389/FCIMB.2021.822562/BIBTEX.

26. Sausset, R., Petit, M.A., Gaboriau-Routhiau, V., and De Paepe, M. (2020). New insights into intestinal phages. Mucosal Immunol. *13*, 205–215. https://doi.org/10.1038/S41385-019-0250-5.

27. Shkoporov, A.N., and Hill, C. (2019). Bacteriophages of the Human Gut: The "Known Unknown" of the Microbiome. Cell Host Microbe *25*, 195–209. https://doi.org/10.1016/J.CHOM.2019.01.017.

28. Sutton, T.D.S., Clooney, A.G., Ryan, F.J., Ross, R.P., and Hill, C. (2019). Choice of assembly software has a critical impact on virome characterisation. Microbiome *7*, 12–15. https://doi.org/10.1186/S40168-019-0626-5/FIGURES/4.

29. Shkoporov, A.N., Turkington, C.J., and Hill, C. (2022). Mutualistic interplay between bacteriophages and bacteria in the human gut. Nat. Rev. Microbiol. *20*, 737–749. https://doi.org/10.1038/s41579-022-00755-4.

30. Tiamani, K., Luo, S., Schulz, S., Xue, J., Costa, R., Khan Mirzaei, M., and Deng, L. (2022). The role of virome in the gastrointestinal tract and beyond. FEMS Microbiol. Rev. *46*, fuac027. https://doi.org/10.1093/FEMSRE/FUAC027.

31. Townsend, E.M., Kelly, L., Muscatt, G., Box, J.D., Hargraves, N., Lilley, D., and Jameson, E. (2021). The Human Gut Phageome: Origins and Roles in the Human Gut Microbiome. Front. Cell. Infect. Microbiol. *11*, 643214. https://doi.org/10.3389/FCIMB.2021.643214/BIBTEX.

32. Shen, J., Zhang, J., Mo, L., Li, Y., Li, Y., Li, C., Kuang, X., Tao, Z., Qu, Z., Wu, L., et al. (2023). Large-scale phage cultivation for commensal human gut bacteria. Cell Host Microbe *31*, 665–677.e7. https://doi.org/10.1016/J.CHOM.2023.03.013.

33. Khan Mirzaei, M., Xue, J., Costa, R., Ru, J., Schulz, S., Taranu, Z.E., and Deng, L. (2021). Challenges of Studying the Human Virome - Relevant Emerging Technologies. Trends Microbiol. *29*, 171–181. https://doi.org/10.1016/J.TIM.2020.05.021.

34. Blanco-Míguez, A., Beghini, F., Cumbo, F., McIver, L.J., Thompson, K.N., Zolfo, M., Manghi, P., Dubois, L., Huang, K.D., Thomas, A.M., et al. (2023). Extending and improving metagenomic taxonomic profiling with uncharacterized species using MetaPhlAn 4. Nat. Biotechnol. *41*, 1633–1644. https://doi.org/10.1038/s41587-023-01688-w.

35. Ogilvie, L.A., and Jones, B.V. (2015). The human gut virome: a multifaceted majority. Front. Microbiol. *6*, 918. https://doi.org/10.3389/FMICB.2015.00918.

36. Ru, J., Khan Mirzaei, M., Xue, J., Peng, X., and Deng, L. (2023). ViroProfiler: a containerized bioinformatics pipeline for viral metagenomic data analysis. Gut Microb. *15*, 2192522. https://doi.org/10.1080/19490976.2023.2192522/SUPPL_FILE/KGMI_A_2192522_SM3001.ZIP.

37. Dutilh, B.E., Cassman, N., McNair, K., Sanchez, S.E., Silva, G.G.Z., Boling, L., Barr, J.J., Speth, D.R., Seguritan, V., Aziz, R.K., et al. (2014). A highly abundant bacteriophage discovered in the unknown sequences of human faecal metagenomes. Nat. Commun. *5*, 4498. https://doi.org/10.1038/ncomms5498.

38. Guerin, E., Shkoporov, A., Stockdale, S.R., Clooney, A.G., Ryan, F.J., Sutton, T.D.S., Draper, L.A., Gonzalez-Tortuero, E., Ross, R.P., and Hill, C. (2018). Biology and Taxonomy of crAss-like Bacteriophages, the Most Abundant Virus in the Human Gut. Cell Host Microbe *24*, 653–664.e6. https://doi.org/10.1016/J.CHOM.2018.10.002.

39. Koonin, E.V., and Yutin, N. (2020). The crAss-like Phage Group: How Metagenomics Reshaped the Human Virome. Trends Microbiol. *28*, 349–359. https://doi.org/10.1016/J.TIM.2020.01.010.

40. Honap, T.P., Sankaranarayanan, K., Schnorr, S.L., Ozga, A.T., Warinner, C., and Lewis, C.M. (2020). Biogeographic study of human gut-associated crAssphage suggests impacts from industrialization and recent expansion. PLoS One *15*, e0226930. https://doi.org/10.1371/JOURNAL.PONE.0226930.

41. Cervantes-Echeverría, M., Gallardo-Becerra, L., Cornejo-Granados, F., and Ochoa-Leyva, A. (2023). The Two-Faced Role of crAssphage Subfamilies in Obesity and Metabolic Syndrome: Between Good and Evil. Genes *14*, 139. https://doi.org/10.3390/GENES14010139/S1.

42. Coclet, C., and Roux, S. (2021). Global overview and major challenges of host prediction methods for uncultivated phages. Curr. Opin. Virol. *49*, 117–126. https://doi.org/10.1016/J.COVIRO.2021.05.003.

43. Edwards, R.A., McNair, K., Faust, K., Raes, J., and Dutilh, B.E. (2016). Computational approaches to predict bacteriophage–host relationships. FEMS Microbiol. Rev. *40*, 258–272. https://doi.org/10.1093/FEMSRE/FUV048.

44. Zafar, H., and Saier, M.H. (2021). Gut Bacteroides species in health and disease. Gut Microb. *13*, 1–20. https://doi.org/10.1080/19490976.2020.1848158.

45. Benler, S., Yutin, N., Antipov, D., Rayko, M., Shmakov, S., Gussow, A.B., Pevzner, P., and Koonin, E.V. (2021). Thousands of previously unknown phages discovered in whole-community human gut metagenomes. Microbiome *9*, 78. https://doi.org/10.1186/S40168-021-01017-W.

46. Coenen, A.R., and Weitz, J.S. (2018). Limitations of Correlation-Based Inference in Complex Virus-Microbe Communities. mSystems *3*. https://doi.org/10.1128/MSYSTEMS.00084-18/ASSET/E5F3A2BF-5BDC-4A2C-B53D-350458BB6C7D/ASSETS/GRAPHIC/SYS0041822540006.JPEG.

47. Weiss, S., Van Treuren, W., Lozupone, C., Faust, K., Friedman, J., Deng, Y., Xia, L.C., Xu, Z.Z., Ursell, L., Alm, E.J., et al. (2016). Correlation detection strategies in microbial data sets vary widely in sensitivity and precision. ISME J. *10*, 1669–1681. https://doi.org/10.1038/ismej.2015.235.

48. Biazzo, M., and Deidda, G. (2022). Fecal Microbiota Transplantation as New Therapeutic Avenue for Human Diseases. J. Clin. Med. *11*, 4119. https://doi.org/10.3390/JCM11144119.

49. Raeisi, H., Noori, M., Azimirad, M., Mohebbi, S.R., Asadzadeh Aghdaei, H., Yadegar, A., and Zali, M.R. (2023). Emerging applications of phage therapy and fecal virome transplantation for treatment of Clostridioides difficile infection: challenges and perspectives. Gut Pathog. *15*, 21. https://doi.org/10.1186/S13099-023-00550-3.

50. Lam, S., Bai, X., Shkoporov, A.N., Park, H., Wu, X., Lan, P., and Zuo, T. (2022). Roles of the gut virome and mycobiome in faecal microbiota transplantation. Lancet. Gastroenterol. Hepatol. *7*, 472–484. https://doi.org/10.1016/S2468-1253(21)00303-4.

51. Mao, X., Larsen, S.B., Zachariassen, L.S.F., Brunse, A., Adamberg, S., Castro Mejia, J.L., Adamberg, K., Nielsen, D.S., Hansen, A.K., Friis Hansen, C.H., and Rasmussen, T.S. (2023). Transfer of modified fecal viromes alleviates symptoms of non-alcoholic liver disease and improve blood glucose regulation in an obesity mouse model. Preprint at bioRxiv. https://doi.org/10.1101/2023.03.20.532903.

52. Liu, Q., Xu, Z., Dai, M., Su, Q., Leung Chan, F.K., and Ng, S.C. (2023). Faecal microbiota transplantations and the role of bacteriophages. Clin. Microbiol. Infect. *29*, 689–694. https://doi.org/10.1016/J.CMI.2022.11.012.

53. Górski, A., Miedzybrodzki, R., and Borysowski, J. (2019). Phage Therapy: A Practical Approach (Springer International Publishing). https://doi.org/10.1007/978-3-030-26736-0/COVER.

54. Lin, D., Lin, H.C., Lin, D., and Lin, H.C. (2021). Fecal Virome Transplantation. Bacteriophages in Therapeutics. https://doi.org/10.5772/INTECHOPEN.95469.

55. Shkoporov, A.N., Ryan, F.J., Draper, L.A., Forde, A., Stockdale, S.R., Daly, K.M., McDonnell, S.A., Nolan, J.A., Sutton, T.D.S., Dalmasso, M., et al. (2018). Reproducible protocols for metagenomic analysis of human faecal phageomes. Microbiome *6*, 68. https://doi.org/10.1186/S40168-018-0446-Z.

56. Jones, J.D., Trippett, C., Suleman, M., Clokie, M.R.J., and Clark, J.R. (2023). The Future of Clinical Phage Therapy in the United Kingdom. Viruses *15*, 721. https://doi.org/10.3390/V15030721.

57. Aranaga, C., Pantoja, L.D., Martínez, E.A., and Falco, A. (2022). Phage Therapy in the Era of Multidrug Resistance in Bacteria: A Systematic Review. Int. J. Mol. Sci. *23*, 4577. https://doi.org/10.3390/IJMS23094577.

58. Pinart, M., Dötsch, A., Schlicht, K., Laudes, M., Bouwman, J., Forslund, S.K., Pischon, T., and Nimptsch, K. (2021). Gut Microbiome Composition in Obese and Non-Obese Persons: A Systematic Review and Meta-Analysis. Nutrients *14*, 12. https://doi.org/10.3390/NU14010012.

59. Yan, Q., Gu, Y., Li, X., Yang, W., Jia, L., Chen, C., Han, X., Huang, Y., Zhao, L., Li, P., et al. (2017). Alterations of the Gut Microbiome in Hypertension. Front. Cell. Infect. Microbiol. *7*, 381. https://doi.org/10.3389/FCIMB.2017.00381.

60. Jie, Z., Xia, H., Zhong, S.L., Feng, Q., Li, S., Liang, S., Zhong, H., Liu, Z., Gao, Y., Zhao, H., et al. (2017). The gut microbiome in atherosclerotic cardiovascular disease. Nat. Commun. *8*, 845. https://doi.org/10.1038/s41467-017-00900-1.

61. Caussy, C., Tripathi, A., Humphrey, G., Bassirian, S., Singh, S., Faulkner, C., Bettencourt, R., Rizo, E., Richards, L., Xu, Z.Z., et al. (2019). A gut microbiome signature for cirrhosis due to nonalcoholic fatty liver disease. Nat. Commun. *10*, 1406. https://doi.org/10.1038/s41467-019-09455-9.

62. Febvre, H.P., Rao, S., Gindin, M., Goodwin, N.D.M., Finer, E., Vivanco, J.S., Lu, S., Manter, D.K., Wallace, T.C., and Weir, T.L. (2019). PHAGE Study: Effects of Supplemental Bacteriophage Intake on Inflammation and Gut Microbiota in Healthy Adults. Nutrients *11*, 666. https://doi.org/10.3390/NU11030666.

63. Belizário, J.E., and Faintuch, J. (2018). Microbiome and Gut Dysbiosis. Exper. Suppl. (Basel) *109*, 459–476. https://doi.org/10.1007/978-3-319-74932-7_13.

64. Pires, D.P., Costa, A.R., Pinto, G., Meneses, L., and Azeredo, J. (2020). Current challenges and future opportunities of phage therapy. FEMS Microbiol. Rev. *44*, 684–700. https://doi.org/10.1093/FEMSRE/FUAA017.

65. De Vos, W.M., Tilg, H., Van Hul, M., and Cani, P.D. (2022). Gut microbiome and health: mechanistic insights. Gut *71*, 1020–1032. https://doi.org/10.1136/GUTJNL-2021-326789.

66. Qin, J., Li, Y., Cai, Z., Li, S., Zhu, J., Zhang, F., Liang, S., Zhang, W., Guan, Y., Shen, D., et al. (2012). A metagenome-wide association study of gut microbiota in type 2 diabetes. Nature *490*, 55–60. https://doi.org/10.1038/nature11450.

67. Nogal, A., Louca, P., Zhang, X., Wells, P.M., Steves, C.J., Spector, T.D., Falchi, M., Valdes, A.M., and Menni, C. (2021). Circulating Levels of the Short-Chain Fatty Acid Acetate Mediate the Effect of the Gut Microbiome on Visceral Fat. Front. Microbiol. *12*, 711359. https://doi.org/10.3389/FMICB.2021.711359/FULL.

68. Bikel, S., López-Leal, G., Cornejo-Granados, F., Gallardo-Becerra, L., García-López, R., Sánchez, F., Equihua-Medina, E., Ochoa-Romo, J.P., López-Contreras, B.E., Canizales-Quinteros, S., et al. (2021). Gut dsDNA virome shows diversity and richness alterations associated with childhood obesity and metabolic syndrome. iScience *24*, 102900. https://doi.org/10.1016/J.ISCI.2021.102900.

CellPress
OPEN ACCESS

69. Sandoval-Vargas, D., Concha-Rubio, N.D., Navarrete, P., Castro, M., and Medina, D.A. (2021). Short communication: Obesity intervention resulting in significant changes in the human gut viral composition. Appl. Sci. *11*, 10039. https://doi.org/10.3390/APP112110039/S1.

70. Kuhn, J.H. (2021). Virus Taxonomy. In Encyclopedia of Virology, pp. 28–37. https://doi.org/10.1016/B978-0-12-809633-8.21231-4.

71. Nishijima, S., Nagata, N., Kiguchi, Y., Kojima, Y., Miyoshi-Akiyama, T., Kimura, M., Ohsugi, M., Ueki, K., Oka, S., Mizokami, M., et al. (2022). Extensive gut virome variation and its associations with host and environmental factors in a population-level cohort. Nat. Commun. *13*, 5252. https://doi.org/10.1038/s41467-022-32832-w.

72. Chen, Q., Ma, X., Li, C., Shen, Y., Zhu, W., Zhang, Y., Guo, X., Zhou, J., and Liu, C. (2020). Enteric Phageome Alterations in Patients With Type 2 Diabetes. Front. Cell. Infect. Microbiol. *10*, 575084. https://doi.org/10.3389/FCIMB.2020.575084.

73. Ma, Y., You, X., Mai, G., Tokuyasu, T., and Liu, C. (2018). A human gut phage catalog correlates the gut phageome with type 2 diabetes. Microbiome *6*, 24. https://doi.org/10.1186/S40168-018-0410-Y/FIGURES/6.

74. Fan, G., Cao, F., Kuang, T., Yi, H., Zhao, C., Wang, L., Peng, J., Zhuang, Z., Xu, T., Luo, Y., et al. (2023). Alterations in the gut virome are associated with type 2 diabetes and diabetic nephropathy. Gut Microb. *15*, 2226925.

75. Bannazadeh Baghi, H., Naghili, B., Shanehbandi, D., and Ebrahimzadeh Leylabadlo, H. (2022). Evaluation of a human gut-associated phage and gut dominant microbial phyla in the metabolic syndrome. Clin. Nutr. ESPEN *50*, 133–137. https://doi.org/10.1016/J.CLNESP.2022.06.009.

76. Han, M., Yang, P., Zhong, C., and Ning, K. (2018). The Human Gut Virome in Hypertension. Front. Microbiol. *9*, 3150. https://doi.org/10.3389/FMICB.2018.03150/FULL.

77. Brunt, E.M., Kleiner, D.E., Wilson, L.A., Belt, P., and Neuschwander-Tetri, B.A.; NASH Clinical Research Network CRN (2011). The NAS and The Histopathologic Diagnosis in NAFLD: Distinct Clinicopathologic Meanings. Hepatology *53*, 810–820. https://doi.org/10.1002/HEP.24127.

78. Metzger, R.N., Krug, A.B., and Eisenächer, K. (2018). Enteric Virome Sensing—Its Role in Intestinal Homeostasis and Immunity. Viruses *10*, 146. https://doi.org/10.3390/V10040146.

79. Clinton, N.A., Hameed, S.A., Agyei, E.K., Jacob, J.C., Oyebanji, V.O., and Jabea, C.E. (2022). Crosstalk between the Intestinal Virome and Other Components of the Microbiota, and Its Effect on Intestinal Mucosal Response and Diseases. J. Immunol. Res. *2022*, 7883945. https://doi.org/10.1155/2022/7883945.

80. Dickson, K., and Lehmann, C. (2019). Inflammatory Response to Different Toxins in Experimental Sepsis Models. Int. J. Mol. Sci. *20*, 4341. https://doi.org/10.3390/IJMS20184341.

81. Kortright, K.E., Chan, B.K., Koff, J.L., and Turner, P.E. (2019). Phage Therapy: A Renewed Approach to Combat Antibiotic-Resistant Bacteria. Cell Host Microbe *25*, 219–232. https://doi.org/10.1016/J.CHOM.2019.01.014.

82. Kitaya, K., and Yasuo, T. (2023). Commonalities and Disparities between Endometriosis and Chronic Endometritis: Therapeutic Potential of Novel Antibiotic Treatment Strategy against Ectopic Endometrium. Int. J. Mol. Sci. *24*, 2059. https://doi.org/10.3390/IJMS24032059.

83. Cicinelli, E., Matteo, M., Tinelli, R., Pinto, V., Marinaccio, M., Indraccolo, U., De Ziegler, D., and Resta, L. (2014). Chronic endometritis due to common bacteria is prevalent in women with recurrent miscarriage as confirmed by improved pregnancy outcome after antibiotic treatment. Reprod. Sci. *21*, 640–647. https://doi.org/10.1177/1933719113508817/METRICS.

84. Koziel, J., and Potempa, J. (2022). Pros and cons of causative association between periodontitis and rheumatoid arthritis. Periodontol *89*, 83–98. https://doi.org/10.1111/PRD.12432.

85. González-Febles, J., and Sanz, M. (2021). Periodontitis and rheumatoid arthritis: What have we learned about their connection and their treatment? Periodontol *87*, 181–203. https://doi.org/10.1111/PRD.12385.

Cell Reports, Volume *43*

Supplemental information

Bacteriophages, gut bacteria,

and microbial pathways

interplay in cardiometabolic health

Daniel Kirk, Ricardo Costeira, Alessia Visconti, Mohammadali Khan Mirzaei, Li Deng, Ana M. Valdes, and Cristina Menni

Supplementary Material

Contents

Table S1: Bacteria at different taxonomic ranks and their alterations across various cardiometabolic diseases, with corresponding references.

Table S2: Microbially derived metabolites, bacteria associated with their production, and their association with cardiometabolic diseases.

Table S3: Association between the human gut phageome and CMD.

Supplementary References

Table S1: [Bacteria at different taxonomic ranks and their alterations across various cardiometabolic diseases, with corresponding references]. Related to Figure 3A.

Bacteria	Obesity	Type 2 Diabetes	Hypertension	CVD	NAFLD
**Phylum**					
Bacteroidetes	↓[S1,S2]			↓[S3]	
Firmicutes	↑[S2]	↓[S4,S5]	↑[S6]		↑[S7]
Proteobacteria	↑[S2]				↑[S8]
**Family**					
Enterobacteriaceae		↑[S9]		↑[S10,S11]	↑[S8]
Lachnospiraceae				↓[S11,S12]	
Ruminococcaceae			↓[S13]	↓[S11,S12,S14]	↓[S15]
Veillonellaceae				↑[S16]	
**Genera**					
Bacteroides	↑↓[S17]	↓[S18,S19]		↓[S10]	
Bifidobacterium	↑↓[S2,S17]	↓[S19]	↓[S20]		
Blautia					↑[S21]
Christensenella			↑[S22]		
Citrobacter		↑[S9]			↑[S9]
Clostridium		↑↓ [S5,S23,S24]	↑↓[S22,S25]		↑[S9,S21]
Collinsella				↑[S16,S26]	↑[S9,S15,S27]
Coprococcus			↓[S20]		↓[S8,S21]
Dorea	↑[S17]				↑[S7,S8,S28]
Enterococcus			↓[S22]	↑[S16,S29]	
Escherichia-Shigella	↑[S17]	↑[S9,S30]		↑[S10,S29]	↑[S8,S9,S21]
Eubacterium	↑[S17]			↓[S26]	↓[S8]
Eggerthella	↓[S17]		↑[S25]		

Faecalibacterium				↓ [S10,S16,S29]	↓ [S8,S21,S27,S31]
Fusobacterium	↑[S1,S17]				
Klebsiella			↑ [S13,S20,S25,S32]	↑ [S10,S12]	
Lactobacillus	↑ [S1,S2]	↑↓[S19]	↓ [S6,S13,S22]	↑ [S3,S16]	
Lactococcus			↓[S22]		↑[S31]
Oscillibacter			↑↓ [S20,S22,S33]		↑ [S9]
Parabacteroides			↑ [S13,S22,S25]		
Porphyromonas			↑[S20]		
Prevotella	↑[S17]		↑[S13,S20]		
Roseburia	↑[S17]	↓ [S5,S19,S23]	↓ [S20,S25,S32]	↓ [S10,S26,S29]	↓ [S7,S21]
Ruminiclostridium 6			↓[S34]		
Salmonella			↑[S25]		
Sporobacter			↓[S33]		
Staphylococcus			↓[S22]		
Streptococcus	↑[S17]		↑[S25,S32]	↑[S10]	↑[S21,S27,S31]
Veillonella			↑[S33]	↑[S11,S12]	
**Species**					
Akkermansia muciniphila	↑[S1,S2]	↓[S19]			
Bacteroides caccae		↑[S23]			
Bacteroides faecichinchillae	↓[S35]				
Bacteroides thetaiotaomicron	↓[S35]				
Bacteroides vulgatus	↓[S2]	↑[S36]			
Blautia hydrogenotorophica	↑[S35]				
Blautia wexlerae	↓[S35]				
Collinsella aerofaciens				↓[S14]	
Coprococcus catus	↑[S35]				
Eggerthella lenta				↑[S10]	
Eubacterium eligens		↓[S5]			

Eubacterium rectale		↓[S23]		↓[S29]	
Eubacterium ventriosum	↑[S35]				
Faecalibacterium prausnitzii	↓[S1]	↓[S5,S19,S23]	↓[S20,S25]	↓[S10]	↓[S31]
Flavonifractor plautii	↓[S35]				
Lactobacillus plantarum	↓[S1]				
Lactobacillus paracasei	↓[S1]				
Prevotella copri		↑[S28]		↓[S10]	↑[S27]
Roseburia intestinalis				↓[S10]	
Ruminococcus bromii	↑[S35]				
Ruminococcus gnavus		↑[S28]			
Ruminococcus obeum	↑[S35]				

Table S2: **[Microbially derived metabolites, bacteria associated with their production, and their association with cardiometabolic diseases].** Related to Figure 3B.

Microbially-derived metabolite	Effect on CMD	Associated CMDs	Associated Bacteria	References
Short-chain fatty acids	Beneficial	Obesity, T2D, NAFLD, hypertension, CVD	**Butyrate**: *Roseburia, Anaerostipe, Faecalibacterium prausnitzii, Eubacterium hallii, Eubacterium rectale, Clostridium leptum*<br><br>**Acetate**: *Bifidobacterium †, Prevotella, Ruminococcus †, Bacteroides †, Clostridium †, Streptococcus †, Akkermansia muciniphila, Blautia hydrogenotrophica*<br><br>**Propionate**: Negativicutes (Firmicutes), *Bacteroides †, Ruminococcus †, Blautia, Akkermansia mucinihila †, Coprococcus catus, Roseburia inulinivorans*	[S37–S39]
Trimethylamine	Harmful	CVD	**TMA**: *Anaerococcus hydrogenalis, Clostridium asparagiforme, Clostridium hathewayi, Clostridium sporogenes, Edwardsiella tarda, Escherichia fergusonii, Proteus penneri, Providencia rettgeri*	[S40]
Secondary bile acids	Harmful	T2D, NAFLD CVD	**Deconjugation**: *Bacteroides †, Bifidobacterium †, Lactobacillus †, Clostridium †, Enterococcus †, Listeria †, Stenotrophomonas, Brucella*<br><br>**Dehydroxylation**: *Clostridium scindens, Clostridium*	[S41]

			hylemonae, Peptacetobacter hiranonis<br><br>**Oxidation & epimerization**: *Ruminococcus gnavus, Clostridium absonum, Stenotrophomonas maltophilia, Collinsella aerofaciens †, Blautia producta, Eggerthella lenta, Clostridium baratii, Enterorhabdus mucosicola*	
Branched chain amino acids	Harmful	Obesity, T2D, CVD	**BCAA**: *Clostridium †, Peptostreptococci, Streptococcus †, Prevotella, Bacteroides †, Klebsiella †, Escherichia coli, Selenomonas ruminantium, Megasphaera elsdenii, Staphylococcus aureus*	[S42]
Aromatic amino acids	Mostly harmful	T2D, NAFLD CVD	**p-cresol**: *Coriobacteriaceae, Clostridium clusters XI and XIVa †*<br><br>**Indole**: *Shigella †, Escherichia coli, Escherichia faecalis, Klebsiella planticola, Proteus vulgaris, other pathogenic bacteria*<br><br>**Phenylacetylglutamine**: *Bacteroides thetaiotaomicron, Proteus mirabilis, Clostridium sporogenes*	[S43,S44]
Lipopolysaccharides	Mostly harmful	Obesity, T2D, NAFLD, CVD	**LPS**: Gram-negative bacteria†	[S45,S46]

Table S3: **[Association between the human gut phageome and CMD].** Related to Figure 4**.** The equals symbol (=) means no change; brackets around an arrow means low significance. Abbreviations: ACVD, atherosclerotic cardiovascular disease; CRP, C-reactive protein; IBS, irritable bowel syndrome; MetS, metabolic syndrome; RCT, randomised controlled trial; T2D, type 2 diabetes.

**Metabolic Disease**	**Subjects**	**Study Design**	**Techniques**	**Phageome Diversity**	**Key Findings**	**Author , Date**
Obesity, MetS	School Children	**Case-Control**<br><br>10 Obesity<br><br>8 MetS<br><br>10 Normal-weight	VLP	(↑)	Significantly different contigs between each group<br><br>↓ highly prevalent (>80%) phage contigs in the normo-weight group were reduced in disease states, going from an average prevalence of 91.54% (normo-weight) to 76.35% (obesity) to 68.27% (MetS)<br><br>Several disease-specific contigs correlate with bacterial taxa and bacterial abundances<br><br>Four highly abundant (present in 80% of all samples) contigs correlated with bacteria: *Collinsella aerofaciens, Parabacteroides distasoni, Phascolarctobacterium sp., Erysipelotrichaceae* family<br><br>Several highly abundant phage contigs correlated with BMI, waist circumference, glucose, triglycerides, and HDL, LDL, and total cholesterol	[S47]
Obesity, MetS	School Children	**Case-Control**	VLP	↓ (only crAssphage was assessed)	↓ prevalence, relative abundance, of crAssphage Alpha sub-family in MetS	[S48]

		10 Obesity<br>8 MetS<br>10 Normal-weight			↑ relative abundance of crAssphage Beta sub-family in MetS<br>↓ in putative crAssphage hosts Bacteroidetes, Bacteroidia, and Bacteroidales<br>↑ in putative crAssphage host *Collinsella*<br>Negative association between *Bacili* and crAssphage abundance	
MetS	Adults	**Case-Control**<br>97 MetS<br>99 Controls	WGS and VLP	↓	↓intracellular phage:bacteria ratios (↓lysogenic phages, ↑ lytic phages?)<br>↓*Crassvirales* phage prevalence in MetS<br>↑phages infecting *Streptococcaceae* (*Streptococcus salivarius*, *Streptococcus sp. HMSC078H03*) and *Bacteroidaceae* (*Bacteroides sp. 3_1_40A, Bacteroides sp. 2_1_16*) in MetS<br>↓phages infecting *Bifidobacteriaceae* (*Bifidobacterium longum, Bifidobacterium adolescentis, Bifidobacterium brevum*) in MetS<br>Prophages confer functions and alter metabolism of bacterial hosts<br>↑ phage richness correlates with ↓ obesity, blood glucose, blood pressure, and triglycerides<br>Phageome changes correlate with bacterial population changes	[S49]
MetS	Adults	**Case-Control**<br>30 MetS	qPCR	N/A	↑ Relative abundance of crAssphage in MetS	[S50]

		30 Control				
Obesity, T2D	Adults	**Case-Control**<br><br>128 Obese (including 74 T2D)<br><br>101 Controls	VLP	↓ (Hong Kong cohort)<br><br>= (Kunming cohort)	↓gut phage-bacterial transkingdom correlations in obesity versus lean controls<br><br>Differentially abundant phages in each group identified, including ↑ *Cellulophaga phage* and *Bacteroides phage* and ↓ *Thermoanaerobicbacterium phage*, *Verrucomicrobia phage* and *Proteus phage* in obese with T2D versus lean control<br><br>Phageome was more severely perturbed in obese with T2D versus obese without T2D<br><br>↓ positive correlations between *Enterobacteria* and *Pseudomonas* phages and healthy-associated bacteria, such as *Faecalibacterium prausnitzii* and *Roseburia intestinalis* in obesity and T2D.	[S51]
T2D	Adults	**Case-Control**<br><br>148 T2D<br><br>290 Non-T2D	WGS	N/A	= proportion of phages genes between T2D, healthy controls, and the other diseases studied (liver cirrhosis, IBS, Crohn's disease, colorectal cancer)<br><br>↓phage-bacteria interactions in T2D<br><br>= in phage function enrichment between disease and healthy<br><br>Differentially abundant phages between healthy versus any of the disease states No differentially abundant phages between each of the disease states	[S52]
T2D	Adults	**Case-Control**	VLP	=	= in abundance of phage families between T2D and healthy control	[S53]

		17 T2D<br><br>29 Controls			Differentially abundant phages in each group identified, including ↑ *Enterobacteria phage cdtl, Enterobacteria phage ES18, Klebsiella phage KP34,* and *Salmonella phage ST64T*, and ↓in *Brochothrix phage A9*, *Brochothrix phage NF5*, *Enterococcus phage phiFL2A,* and *Salmonella phage* PVP-SE1 in T2D.<br><br>↑relative abundance of gram-negative phages in T2D<br><br>↑*Enterobacteriaceae* and their phages in T2D<br><br>Correlations between phages and T2D clinical markers (fasting blood glucose and insulin, insulin 0.5 h and 2 h after a meal, highly sensitive CRP, and free thyroxine)	
T2D	Adults	**Case-Control**<br><br>71 T2D<br><br>74 Controls	WGS and VLP	=	↑number of phages in T2D<br><br>↑relative abundance of core (present in >66.6% of the samples) phage OTUs in T2D<br><br>*Escherichia* and *Bacteroides* genera had the highest number of interactions with phages<br><br>Abundance of phages did not correlate with the abundances of their inferred bacterial hosts	[S54]
T2D	Adults	**Case-Control**<br><br>90 T2D (of which 49 had neuropathy)<br><br>42 Controls	VLP	↓	Differentially abundant viral taxa (6), families (12) and species (81), of which 78% were phages<br><br>↓ *Flavobacterium, Cellulophaga, Staphylococcus , Synechoccus , Curvibacter , Clostridoides , Tenacibaculum , Paenibacillus , Lactobacillus, Listeria,* and *Citrobacter* phages, amongst others, in T2D	[S55]

					↑*Shigella* and *Xylella* phages in T2D<br><br>Loss of diverse viral functions in T2D, including viral replication and integration and phage lysis of bacteria host<br><br>Viral and bacterial diversity were positively correlated in T2D but not in controls<br><br>Alterations in transkingdom interactions, including both loss and gain of phage-bacteria interactions in T2D<br><br>↑positive viral-bacteria correlations and ↓negative viral-bacteria correlations in T2D<br><br>Correlation between *Streptococcus satellite phage* and five bacteria, including *Collinsella aerofaciens,* and *Pseudomonas phage* and four bacteria, including *Ruminococcus bromii* and *Akkermansia muciniphila*, lost in T2D	
Hypertension & prehypertension	Adults	**Case-Control**<br><br>99 Hypertensives<br><br>56 Pre-hypertensives<br><br>41 Controls	WGS	=	Dominant phages were different across each group, including:<br><br>• ↑ *Klebsiella phage KP32, Cyanophage S-TIM5, and Salmonella phage FSL SP-004* in hypertension*;*<br>• ↑ *Cronobacter phage CR3, Cronobacter phage ENT39118,* and *Cronobacter phage phiES15* in pre-hypertension;<br>• ↑ *Salmonella phage vB-SemP-Emek, Pseudomonas phage PaMx11* and *Gordonia phage GTE8* in controls<br><br>Phage-bacterial interactions altered in hypertension	[S56]
NAFLD	Adults	**Case-Control**<br><br>73 NAFLD (29 NAS Score 0-4,	VLP	↓	↓ proportion of phages in advanced NAFLD	[S57]

		44 NAS score 5-8, 37 F0–F1 fibrosis, 36 F2–F4 fibrosis)<br>13 Mild primary biliary cholangitis<br>22 Controls			↑*Leuconostoc phages* were associated with a ↓BMI, lower blood glucose and HbA1c levels, and ↑*Escherichia* and *Enterobacteria* phages were associated with ↑blood glucose levels	
ACVD, T2D, obesity	Adults 40-80 years old	Case-Control<br>218 ACVD, 187 Controls<br>171 T2D, 174 Controls<br>72 Obesity, 79 Controls	WGS	N/A	↑Mycobacterium phages Astraea, Gizmo, Pleione, ScottMcG, Spud, MoMoMixon, Aeromonas phage, Brochothrix phage A9, Synechococcus phage S-CAM1 in both CVD and obesity<br>↑*Mycobacterium phage Gumball* and *Escherichia phage wV7* in both CVD and T2D<br>Known hosts for phages enriched in ACVD were mainly of the family *Enterobacteriaceae* and *Streptococcus* genus.	[S10]
T2D, hypertension, obesity, age	Adults	**Cohort**<br>4198 Total (including 642 T2D)	WGS	↑ (with age)	Phageome composition associated with age hypertension, diabetes, and medication to treat metabolic conditions.<br>dsDNA phageome profile diversity was associated with age and diabetes medication use but not hypertension or diabetes.	[S58]
MetS	Adult males	**RCT, longitudinal**	VLP	↓ in non-responders versus	Phages unique to MetS replaced by new phages	[S59]

		(faecal transplant)<br><br>6 recipients, 5 donors		responders after treatment	Differentially abundant phages in responders versus non-responders post-treatment<br><br>phages acquired via treatment correlate with percentage increase of glucose disposal rate	
Obesity	Adults	**Intervention** (various obesity interventions)<br><br>21 pre-treatment and 28 post-treatment	WGS	↓ pre- versus post-obesity treatment	↑*Uncultured crAssphage*, *Enterobacteria phage AA91-ss* and *Enterobacteria phage 88* pre-obesity treatment<br><br>↑ *Enterobacteria phages HK629, lambda and EP460, Escherichia phages pro 147* and *483,* and *Yersinia phage L14_C*., amongst others post-obesity treatment	[S60]

Supplementary References

S1. Crovesy, L., Masterson, D., and Rosado, E.L. (2020). Profile of the gut microbiota of adults with obesity: a systematic review. Eur J Clin Nutr *74*, 1251–1262. 10.1038/S41430-020-0607-6.

S2. Rinninella, E., Raoul, P., Cintoni, M., Franceschi, F., Miggiano, G.A.D., Gasbarrini, A., and Mele, M.C. (2019). What is the Healthy Gut Microbiota Composition? A Changing Ecosystem across Age, Environment, Diet, and Diseases. Microorganisms 2019, Vol. 7, Page 14 *7*, 14. 10.3390/MICROORGANISMS7010014.

S3. Yamashita, T., Emoto, T., Sasaki, N., and Hirata, K.I. (2016). Gut Microbiota and Coronary Artery Disease. Int Heart J *57*, 663–671. 10.1536/IHJ.16-414.

S4. Larsen, N., Vogensen, F.K., Van Den Berg, F.W.J., Nielsen, D.S., Andreasen, A.S., Pedersen, B.K., Al-Soud, W.A., Sørensen, S.J., Hansen, L.H., and Jakobsen, M. (2010). Gut microbiota in human adults with type 2 diabetes differs from non-diabetic adults. PLoS One *5*. 10.1371/JOURNAL.PONE.0009085.

S5. Karlsson, F.H., Tremaroli, V., Nookaew, I., Bergström, G., Behre, C.J., Fagerberg, B., Nielsen, J., and Bäckhed, F. (2013). Gut metagenome in European women with normal, impaired and diabetic glucose control. Nature *498*, 99–103. 10.1038/NATURE12198.

S6. Palmu, J., Salosensaari, A., Havulinna, A.S., Cheng, S., Inouye, M., Jain, M., Salido, R.A., Sanders, K., Brennan, C., Humphrey, G.C., et al. (2020). Association between the gut microbiota and blood pressure in a population cohort of 6953 individuals. J Am Heart Assoc *9*, 16641. 10.1161/JAHA.120.016641.

S7. Raman, M., Ahmed, I., Gillevet, P.M., Probert, C.S., Ratcliffe, N.M., Smith, S., Greenwood, R., Sikaroodi, M., Lam, V., Crotty, P., et al. (2013). Fecal Microbiome and Volatile Organic Compound Metabolome in Obese Humans With Nonalcoholic Fatty Liver Disease. Clinical Gastroenterology and Hepatology *11*, 868-875.e3. 10.1016/J.CGH.2013.02.015.

S8. Vijay, A., and Valdes, A.M. (2021). Role of the gut microbiome in chronic diseases: a narrative review. European Journal of Clinical Nutrition 2021 76:4 *76*, 489–501. 10.1038/s41430-021-00991-6.

S9. Frost, F., Kacprowski, T., Rühlemann, M., Pietzner, M., Bang, C., Franke, A., Nauck, M., Völker, U., Völzke, H., Dörr, M., et al. (2021). Long-term instability of the intestinal microbiome is associated with metabolic liver disease, low microbiota diversity, diabetes mellitus and impaired exocrine pancreatic function. Gut *70*, 522–530. 10.1136/GUTJNL-2020-322753.

S10. Jie, Z., Xia, H., Zhong, S.L., Feng, Q., Li, S., Liang, S., Zhong, H., Liu, Z., Gao, Y., Zhao, H., et al. (2017). The gut microbiome in atherosclerotic cardiovascular disease. Nature Communications 2017 8:1 *8*, 1–12. 10.1038/s41467-017-00900-1.

S11. Zeng, X., Gao, X., Peng, Y., Wu, Q., Zhu, J., Tan, C., Xia, G., You, C., Xu, R., Pan, S., et al. (2019). Higher Risk of Stroke Is Correlated With Increased Opportunistic Pathogen Load and Reduced Levels of Butyrate-Producing Bacteria in the Gut. Front Cell Infect Microbiol *9*. 10.3389/FCIMB.2019.00004.

S12. Liu, H., Chen, X., Hu, X., Niu, H., Tian, R., Wang, H., Pang, H., Jiang, L., Qiu, B., Chen, X., et al. (2019). Alterations in the gut microbiome and metabolism with coronary artery disease severity. Microbiome *7*, 1–14. 10.1186/S40168-019-0683-9/FIGURES/4.

S13. Masenga, S.K., Hamooya, B., Hangoma, J., Hayumbu, V., Ertuglu, L.A., Ishimwe, J., Rahman, S., Saleem, M., Laffer, C.L., Elijovich, F., et al. (2022). Recent advances in modulation of

cardiovascular diseases by the gut microbiota. Journal of Human Hypertension 2022 36:11 *36*, 952–959. 10.1038/s41371-022-00698-6.

S14. Menni, C., Lin, C., Cecelja, M., Mangino, M., Matey-Hernandez, M.L., Keehn, L., Mohney, R.P., Steves, C.J., Spector, T.D., Kuo, C.F., et al. (2018). Gut microbial diversity is associated with lower arterial stiffness in women. Eur Heart J *39*, 2390a–2397a. 10.1093/EURHEARTJ/EHY226.

S15. Astbury, S., Atallah, E., Vijay, A., Aithal, G.P., Grove, J.I., and Valdes, A.M. (2020). Lower gut microbiome diversity and higher abundance of proinflammatory genus Collinsella are associated with biopsy-proven nonalcoholic steatohepatitis. Gut Microbes *11*, 569–580. 10.1080/19490976.2019.1681861/SUPPL_FILE/KGMI_A_1681861_SM8563.DOCX.

S16. Cui, X., Ye, L., Li, J., Jin, L., Wang, W., Li, S., Bao, M., Wu, S., Li, L., Geng, B., et al. (2018). Metagenomic and metabolomic analyses unveil dysbiosis of gut microbiota in chronic heart failure patients. Sci Rep *8*. 10.1038/S41598-017-18756-2.

S17. Pinart, M., Dötsch, A., Schlicht, K., Laudes, M., Bouwman, J., Forslund, S.K., Pischon, T., and Nimptsch, K. (2021). Gut Microbiome Composition in Obese and Non-Obese Persons: A Systematic Review and Meta-Analysis. Nutrients *14*. 10.3390/NU14010012.

S18. Durack, J., and Lynch, S. V. (2019). The gut microbiome: Relationships with disease and opportunities for therapy. Journal of Experimental Medicine *216*, 20–40. 10.1084/JEM.20180448.

S19. Gurung, M., Li, Z., You, H., Rodrigues, R., Jump, D.B., Morgun, A., and Shulzhenko, N. (2020). Role of gut microbiota in type 2 diabetes pathophysiology. EBioMedicine *51*, 102590. 10.1016/J.EBIOM.2019.11.051.

S20. Li, J., Zhao, F., Wang, Y., Chen, J., Tao, J., Tian, G., Wu, S., Liu, W., Cui, Q., Geng, B., et al. (2017). Gut microbiota dysbiosis contributes to the development of hypertension. Microbiome *5*, 1–19. 10.1186/S40168-016-0222-X/FIGURES/7.

S21. Sharpton, S.R., Ajmera, V., and Loomba, R. (2019). Emerging Role of the Gut Microbiome in Nonalcoholic Fatty Liver Disease: From Composition to Function. Clinical Gastroenterology and Hepatology *17*, 296–306. 10.1016/J.CGH.2018.08.065.

S22. Dan, X., Mushi, Z., Baili, W., Han, L., Enqi, W., Huanhu, Z., and Shuchun, L. (2019). Differential Analysis of Hypertension-Associated Intestinal Microbiota. Int J Med Sci *16*, 872–881. 10.7150/IJMS.29322.

S23. Qin, J., Li, Y., Cai, Z., Li, S., Zhu, J., Zhang, F., Liang, S., Zhang, W., Guan, Y., Shen, D., et al. (2012). A metagenome-wide association study of gut microbiota in type 2 diabetes. Nature 2012 490:7418 *490*, 55–60. 10.1038/nature11450.

S24. Wu, H., Tremaroli, V., Schmidt, C., Lundqvist, A., Olsson, L.M., Krämer, M., Gummesson, A., Perkins, R., Bergström, G., and Bäckhed, F. (2020). The Gut Microbiota in Prediabetes and Diabetes: A Population-Based Cross-Sectional Study. Cell Metab *32*, 379-390.e3. 10.1016/J.CMET.2020.06.011.

S25. Yan, Q., Gu, Y., Li, X., Yang, W., Jia, L., Chen, C., Han, X., Huang, Y., Zhao, L., Li, P., et al. (2017). Alterations of the Gut Microbiome in Hypertension. Front Cell Infect Microbiol *7*. 10.3389/FCIMB.2017.00381.

S26. Karlsson, F.H., Fåk, F., Nookaew, I., Tremaroli, V., Fagerberg, B., Petranovic, D., Bäckhed, F., and Nielsen, J. (2012). Symptomatic atherosclerosis is associated with an altered gut metagenome. Nature Communications 2012 3:1 *3*, 1–8. 10.1038/ncomms2266.

S27. Ruuskanen, M.O., Åberg, F., Männistö, V., Havulinna, A.S., Méric, G., Liu, Y., Loomba, R., Vázquez-Baeza, Y., Tripathi, A., Valsta, L.M., et al. (2021). Links between gut microbiome

composition and fatty liver disease in a large population sample. Gut Microbes *13*, 1–22. 10.1080/19490976.2021.1888673/SUPPL_FILE/KGMI_A_1888673_SM1172.ZIP.

S28. Ruuskanen, M.O., Erawijantari, P.P., Havulinna, A.S., Liu, Y., Méric, G., Tuomilehto, J., Inouye, M., Jousilahti, P., Salomaa, V., Jain, M., et al. (2022). Gut Microbiome Composition Is Predictive of Incident Type 2 Diabetes in a Population Cohort of 5,572 Finnish Adults. Diabetes Care *45*, 811–818. 10.2337/DC21-2358.

S29. Zhu, Q., Gao, R., Zhang, Y., Pan, D., Zhu, Y., Zhang, X., Yang, R., Jiang, R., Xu, Y., and Qin, H. (2018). Dysbiosis signatures of gut microbiota in coronary artery disease. Physiol Genomics *50*, 893–903. 10.1152/PHYSIOLGENOMICS.00070.2018/ASSET/IMAGES/LARGE/ZH70111843130007.JPEG.

S30. Hartstra, A. V., Bouter, K.E.C., Bäckhed, F., and Nieuwdorp, M. (2015). Insights Into the Role of the Microbiome in Obesity and Type 2 Diabetes. Diabetes Care *38*, 159–165. 10.2337/DC14-0769.

S31. Caussy, C., Tripathi, A., Humphrey, G., Bassirian, S., Singh, S., Faulkner, C., Bettencourt, R., Rizo, E., Richards, L., Xu, Z.Z., et al. (2019). A gut microbiome signature for cirrhosis due to nonalcoholic fatty liver disease. Nature Communications 2019 10:1 *10*, 1–9. 10.1038/s41467-019-09455-9.

S32. Verhaar, B.J.H., Collard, D., Prodan, A., Levels, J.H.M., Zwinderman, A.H., Backhed, F., Vogt, L., Peters, M.J.L., Muller, M., Nieuwdorp, M., et al. (2020). Associations between gut microbiota, faecal short-chain fatty acids, and blood pressure across ethnic groups: the HELIUS study. Eur Heart J *41*, 4259–4267. 10.1093/EURHEARTJ/EHAA704.

S33. Sun, S., Lulla, A., Sioda, M., Winglee, K., Wu, M.C., Jacobs, D.R., Shikany, J.M., Lloyd-Jones, D.M., Launer, L.J., Fodor, A.A., et al. (2019). Gut Microbiota Composition and Blood Pressure. Hypertension *73*, 998–1006. 10.1161/HYPERTENSIONAHA.118.12109.

S34. Louca, P., Nogal, A., Wells, P.M., Asnicar, F., Wolf, J., Steves, C.J., Spector, T.D., Segata, N., Berry, S.E., Valdes, A.M., et al. (2021). Gut microbiome diversity and composition is associated with hypertension in women. J Hypertens *39*, 1810–1816. 10.1097/HJH.0000000000002878.

S35. Castaner, O., Goday, A., Park, Y.M., Lee, S.H., Magkos, F., Shiow, S.A.T.E., and Schröder, H. (2018). The Gut Microbiome Profile in Obesity: A Systematic Review. Int J Endocrinol *2018*. 10.1155/2018/4095789.

S36. Pedersen, H.K., Gudmundsdottir, V., Nielsen, H.B., Hyotylainen, T., Nielsen, T., Jensen, B.A.H., Forslund, K., Hildebrand, F., Prifti, E., Falony, G., et al. (2016). Human gut microbes impact host serum metabolome and insulin sensitivity. Nature 2016 535:7612 *535*, 376–381. 10.1038/nature18646.

S37. Venegas, D.P., De La Fuente, M.K., Landskron, G., González, M.J., Quera, R., Dijkstra, G., Harmsen, H.J.M., Faber, K.N., and Hermoso, M.A. (2019). Short Chain Fatty Acids (SCFAs)-Mediated Gut Epithelial and Immune Regulation and Its Relevance for Inflammatory Bowel Diseases. Front Immunol *10*. 10.3389/FIMMU.2019.00277.

S38. Nogal, A., Valdes, A.M., and Menni, C. (2021). The role of short-chain fatty acids in the interplay between gut microbiota and diet in cardio-metabolic health. Gut Microbes *13*, 1–24. 10.1080/19490976.2021.1897212.

S39. Reichardt, N., Duncan, S.H., Young, P., Belenguer, A., McWilliam Leitch, C., Scott, K.P., Flint, H.J., and Louis, P. (2014). Phylogenetic distribution of three pathways for propionate

production within the human gut microbiota. The ISME Journal 2014 8:6 *8*, 1323–1335. 10.1038/ismej.2014.14.

S40. Liu, Y., and Dai, M. (2020). Trimethylamine N-Oxide Generated by the Gut Microbiota Is Associated with Vascular Inflammation: New Insights into Atherosclerosis. Mediators Inflamm *2020*. 10.1155/2020/4634172.

S41. Guzior, D. V., and Quinn, R.A. (2021). Review: microbial transformations of human bile acids. Microbiome *9*, 1–13. 10.1186/S40168-021-01101-1/FIGURES/5.

S42. Gojda, J., and Cahova, M. (2021). Gut Microbiota as the Link between Elevated BCAA Serum Levels and Insulin Resistance. Biomolecules 2021, Vol. 11, Page 1414 *11*, 1414. 10.3390/BIOM11101414.

S43. Saito, Y., Sato, T., Nomoto, K., and Tsuji, H. (2018). Identification of phenol- and p-cresol-producing intestinal bacteria by using media supplemented with tyrosine and its metabolites. FEMS Microbiol Ecol *94*, 125. 10.1093/FEMSEC/FIY125.

S44. Lee, J.H., and Lee, J. (2010). Indole as an intercellular signal in microbial communities. FEMS Microbiol Rev *34*, 426–444. 10.1111/J.1574-6976.2009.00204.X.

S45. Steimle, A., Autenrieth, I.B., and Frick, J.S. (2016). Structure and function: Lipid A modifications in commensals and pathogens. International Journal of Medical Microbiology *306*, 290–301. 10.1016/J.IJMM.2016.03.001.

S46. Di Lorenzo, F., De Castro, C., Silipo, A., and Molinaro, A. (2019). Lipopolysaccharide structures of Gram-negative populations in the gut microbiota and effects on host interactions. FEMS Microbiol Rev *43*, 257–272. 10.1093/FEMSRE/FUZ002.

S47. Bikel, S., López-Leal, G., Cornejo-Granados, F., Gallardo-Becerra, L., García-López, R., Sánchez, F., Equihua-Medina, E., Ochoa-Romo, J.P., López-Contreras, B.E., Canizales-Quinteros, S., et al. (2021). Gut dsDNA virome shows diversity and richness alterations associated with childhood obesity and metabolic syndrome. iScience *24*. 10.1016/J.ISCI.2021.102900.

S48. Cervantes-Echeverría, M., Gallardo-Becerra, L., Cornejo-Granados, F., and Ochoa-Leyva, A. (2023). The Two-Faced Role of crAssphage Subfamilies in Obesity and Metabolic Syndrome: Between Good and Evil. Genes (Basel) *14*, 139. 10.3390/GENES14010139/S1.

S49. de Jonge, P.A., Wortelboer, K., Scheithauer, T.P.M., van den Born, B.J.H., Zwinderman, A.H., Nobrega, F.L., Dutilh, B.E., Nieuwdorp, M., and Herrema, H. (2022). Gut virome profiling identifies a widespread bacteriophage family associated with metabolic syndrome. Nat Commun *13*. 10.1038/S41467-022-31390-5.

S50. Bannazadeh Baghi, H., Naghili, B., Shanehbandi, D., and Ebrahimzadeh Leylabadlo, H. (2022). Evaluation of a human gut-associated phage and gut dominant microbial phyla in the metabolic syndrome. Clin Nutr ESPEN *50*, 133–137. 10.1016/J.CLNESP.2022.06.009.

S51. Yang, K., Niu, J., Zuo, T., Sun, Y., Xu, Z., Tang, W., Liu, Q., Zhang, J., Ng, E.K.W., Wong, S.K.H., et al. (2021). Alterations in the Gut Virome in Obesity and Type 2 Diabetes Mellitus. Gastroenterology *161*, 1257-1269.e13. 10.1053/J.GASTRO.2021.06.056.

S52. Li, M., Wang, C., Guo, Q., Xu, C., Xie, Z., Tan, J., Wu, S., Wang, P., Guo, J., Fang, Z., et al. (2022). More Positive or More Negative? Metagenomic Analysis Reveals Roles of Virome in Human Disease-Related Gut Microbiome. Front Cell Infect Microbiol *12*. 10.3389/FCIMB.2022.846063.

S53. Chen, Q., Ma, X., Li, C., Shen, Y., Zhu, W., Zhang, Y., Guo, X., Zhou, J., and Liu, C. (2021). Enteric Phageome Alterations in Patients With Type 2 Diabetes. Front Cell Infect Microbiol *10*. 10.3389/FCIMB.2020.575084.

S54. Ma, Y., You, X., Mai, G., Tokuyasu, T., and Liu, C. (2018). A human gut phage catalog correlates the gut phageome with type 2 diabetes. Microbiome *6*, 1–12. 10.1186/S40168-018-0410-Y/FIGURES/6.

S55. Fan, G., Cao, F., Kuang, T., Yi, H., Zhao, C., Wang, L., Peng, J., Zhuang, Z., Xu, T., Luo, Y., et al. (2023). Alterations in the gut virome are associated with type 2 diabetes and diabetic nephropathy. Gut Microbes *15*, 2226925. 10.1080/19490976.2023.2226925/SUPPL_FILE/KGMI_A_2226925_SM4471.ZIP.

S56. Han, M., Yang, P., Zhong, C., and Ning, K. (2018). The Human Gut Virome in Hypertension. Front Microbiol *9*. 10.3389/FMICB.2018.03150/FULL.

S57. Lang, S., Demir, M., Martin, A., Jiang, L., Zhang, X., Duan, Y., Gao, B., Wisplinghoff, H., Kasper, P., Roderburg, C., et al. (2020). Intestinal Virome Signature Associated With Severity of Nonalcoholic Fatty Liver Disease. Gastroenterology *159*, 1839–1852. 10.1053/J.GASTRO.2020.07.005.

S58. Nishijima, S., Nagata, N., Kiguchi, Y., Kojima, Y., Miyoshi-Akiyama, T., Kimura, M., Ohsugi, M., Ueki, K., Oka, S., Mizokami, M., et al. (2022). Extensive gut virome variation and its associations with host and environmental factors in a population-level cohort. Nature Communications 2022 13:1 *13*, 1–14. 10.1038/s41467-022-32832-w.

S59. Manrique, P., Zhu, Y., van der Oost, J., Herrema, H., Nieuwdorp, M., de Vos, W.M., and Young, M. (2021). Gut bacteriophage dynamics during fecal microbial transplantation in subjects with metabolic syndrome. Gut Microbes *13*, 1–15. 10.1080/19490976.2021.1897217/SUPPL_FILE/KGMI_A_1897217_SM5623.DOCX.

S60. Sandoval-Vargas, D., Concha-Rubio, N.D., Navarrete, P., Castro, M., and Medina, D.A. (2021). Short communication: Obesity intervention resulting in significant changes in the human gut viral composition. Applied Sciences (Switzerland) *11*, 10039. 10.3390/APP112110039/S1.
